# Optimal design and validation of antiviral siRNA for targeting HIV-1

**DOI:** 10.1186/1742-4690-4-80

**Published:** 2007-11-08

**Authors:** Yuki Naito, Kyoko Nohtomi, Toshinari Onogi, Rie Uenishi, Kumiko Ui-Tei, Kaoru Saigo, Yutaka Takebe

**Affiliations:** 1 Department of Biophysics and Biochemistry, Graduate School of Science, University of Tokyo, 7-3-1 Hongo, Bunkyo-ku, Tokyo, 113-0033, Japan; 2 Laboratory of Molecular Virology and Epidemiology, AIDS Research Center, National Institute of Infectious Diseases, 1-23-1 Toyama, Shinjuku-ku, Tokyo, 162-8640, Japan

## Abstract

We propose rational designing of antiviral short-interfering RNA (siRNA) targeting highly divergent HIV-1. In this study, conserved regions within HIV-1 genomes were identified through an exhaustive computational analysis, and the functionality of siRNAs targeting the highest possible conserved regions was validated. We present several promising antiviral siRNA candidates that effectively inhibited multiple subtypes of HIV-1 by targeting the best conserved regions in pandemic HIV-1 group M strains.

## Findings

RNA interference (RNAi) is now widely used to knockdown gene expression in a sequence-specific manner, making it a powerful tool not only for studying gene function, but also for therapeutic applications including antiviral treatments [1,2]. The replication of a wide range of viruses can be successfully inhibited using RNAi with both short interfering RNA (siRNA) and siRNA expression vectors [3,4]. However, for RNA viruses such as HIV-1, designing functional siRNAs that target viral sequences is problematic because of their extraordinarily high genetic diversity. We analyzed 495 entries of near full-length HIV-1 group M sequences available in the Los Alamos HIV Sequence Database, and selected the highest-possible conserved target sites for designing optimal antiviral siRNAs. It is known that RNAi-resistant viral mutants emerge rapidly when targeting viral sequences due to their high mutation rate [5-7]. Since highly conserved sequences are likely to contain structurally or functionally constrained elements, our approach is anticipated to resist viral mutational escape.

**Figure 1:**
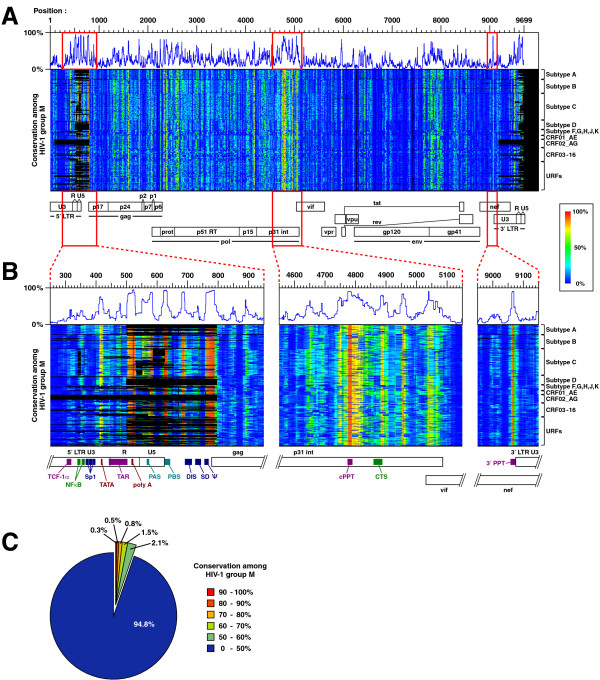
**Conservations of siRNA target sequences among HIV-1 group M**. (**A**) A total of 4,417,157 siRNA targets were generated from the 495 HIV-1 sequences, and their conservations within the HIV-1 genomes are represented using a color density plot. The line plot above the color chart represents the highest value in each position. (**B**) A detailed view of the three conserved regions; 5' LTR, the cPPT/CTS in the integrase gene, and 3' PPT. 'Position' indicates the 5'-most position of each 21-mer. The landmarks of the HIV-1 genome are adjusted to align at the center of the siRNAs by shifting 10 bp to the left. (**C**) Pie chart indicating the percentage of the 4,417,157 siRNA target sites at each conservation level.

First, we performed a detailed analysis on the HIV-1 genome to identify highly conserved targets by using 495 near full-length genome sequences of HIV-1 group M (listed in Additional file 1). Every possible 21-mer was generated from all of the HIV-1 group M sequences, and their conservations among the 495 HIV-1 sequences were exhaustively determined using siVirus engine [8]. We defined 'conservation' as the percentage of sequence entries out of the 495 HIV-1 sequences that showed perfect identity (*i.e*., 21/21 matches) with the cognate 21-mer. Since many of the HIV-1 sequence entries lack 5' untranslated region (5' UTR), the 3' LTR sequence was used to compensate for the lack of 5' LTR sequences in order to avoid underestimating conservation in such regions. For the regions that cannot be compensated for in this way (depicted in Figure 1A and 1B left panel, colored black), conservation was calculated by considering only the HIV-1 sequences that contain the corresponding regions. The result revealed that HIV-1 genomes are not conserved for consecutive 21 bp for the most part, resulting in the poor conservation of many of the 21-mers over the HIV-1 sequences (Figure 1A, colored blue). As shown in Figure 1C, only 5.2% of the possible 21-mers are >50% conserved. Furthermore, highly (>70%) conserved 21-mers constitute only 1.6% of all 21-mers. It is of note that many of the published anti-HIV-1 siRNA sequences do not fall into this 'highly conserved' category (Additional file 2 and [9]). From these results, we anticipate that most of the possible siRNAs are not suitable for the efficient targeting of HIV-1.

However, our analysis has identified several distinct regions that are highly conserved in the HIV-1 genome (Figure 1B). Such regions include the regulatory domains responsible for the viral gene expression, such as the TATA sequence and polyadenylation signal (AAUAAA). In addition, several regions essential for the regulation of viral replication were also highly conserved, including the primer activation signal (PAS)[10], primer binding site (PBS), packaging signal (Ψ), central polypurine tract (cPPT), central termination sequence (CTS), and 3' polypurine tract (3' PPT). All of these highly conserved sequences are constrained at the nucleotide sequence level or by their RNA secondary structure in order to execute their functions. In contrast, regions constrained by amino acid sequences were not necessarily conserved at the nucleotide sequence level due to the wobbling of the third base in the codon (data not shown). siRNAs targeting the highly conserved regions are expected to overwhelm the high level of sequence diversity of the HIV-1 genome, and also to reduce the chances of viral mutational escapes.

Total of 216 highly conserved (>70%) siRNA targets identified in this study are listed in Additional file 3. In mammalian RNAi, the efficacy of each siRNA varies markedly depending on its sequence. According to our guidelines for the selection of effective siRNAs [11,12], 31 out of 216 siRNAs were predicted to be functional. Similarly, 30 and 44 siRNAs are functional according to the algorithms reported by Reynolds *et al*. [13], and Amarzguioui *et al*. [14], respectively (Additional file 3). This suggests that only a limited fraction of 21-mers is best suited for use as functional antiviral siRNAs.

**Figure 2:**
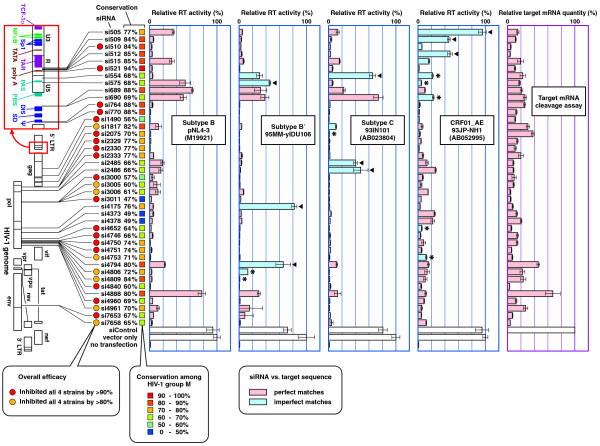
**Validation of 41 siRNAs**. The antiviral efficacy of each siRNA was tested against four HIV-1 infectious molecular clones: pNL4-2 (subtype B); 95MM-yIDU106 (subtype B'); 93IN101 (subtype C); or 93JP-NH1 (CRF01_AE). The potency of each siRNA was tested using the target mRNA cleavage assay (rightmost panel). The ability of each siRNA to cleave its target was evaluated by the target mRNA cleavage assay.

For the functional validation, 23 siRNAs from Additional file 3, and 18 additional siRNAs targeting moderately-conserved regions were selected based on the following criteria: (I) predicted to be functional by the algorithm of Ui-Tei *et al*. [11,12], and (II) the sequence has perfect identity with pNL4-3 (GenBank M19921). The 41 siRNA sequences selected and their target sites are detailed in Additional file 4. We first tested the efficacy of each siRNA using target mRNA cleavage assay (Additional file 5 and [15]). Briefly, a vector expressing reporter mRNA that contains the siRNA target site was cotransfected into HeLa cells with the corresponding siRNA, and the mRNA cleavage activity of the siRNA was evaluated by measuring the quantity of surviving mRNA using real-time RT-PCR. This assay allows us to directly monitor the sequence-dependent potency of siRNA itself, without being affected by the differences in target gene expression level or target secondary structures. The result showed that 39 out of the 41 siRNAs gave >60% silencing at 5 nM (Figure 2, rightmost panel). si4794 and si4888 were not functional, probably due to the long consecutive Gs in si4794 and internal palindromes (AAAAUUUU) in si4888 [11,13].

Next, siRNAs were evaluated for their antiviral efficacy against three evolutionary-distant groups of HIV-1: subtypes B and B' (Thailand variant of subtype B [16]); subtype C; and CRF01_AE. Each siRNA was cotransfected into HeLa cells at 5 nM with one of the four infectious molecular clones: pNL4-3 (subtype B); 95MM-yIDU106 (subtype B'); 93IN101 (subtype C); or 93JP-NH1 (CRF01_AE). Culture supernatants were collected 48 h after transfection and the viral reverse transcriptase activity was measured (Additional file 5 and [17]). The results show that 26 of the 41 siRNAs effectively inhibited viral replication of all four strains by >80% (Figure 2, marked with red or orange circles). Of the remaining 15 siRNAs, 13 of them (except si4794/4888) were shown to be functional in the target mRNA cleavage assay, and 12 of them (except si690/4794/4888) inhibited the replication of at least one viral strain by >80%, indicating that the designed siRNAs have the potential to induce RNAi. In several viral strains, nucleotide substitutions in their target sites essentially abolished the inhibition of viral replication (Figure 2, blue bars with arrowheads). However, mismatches near the ends of the target sites (see Additional file 6) did not necessarily abolish the siRNA efficacy (Figure 2, blue bars with asterisks). si689 and si690 did not inhibit viral replication even though these siRNAs perfectly matched to their target sites (confirmed by DNA sequencing of the infectious molecular clones). This is probably due to the stable secondary structure at the si689-690 target sites in both BMH (branched multiple hairpin) conformation and LDI (long distance interaction) conformation of the HIV-1 leader RNA [18] (see Additional file 4). It should be noted that the efficacy of si575 differed when targeting pNL4-3 and 93IN101. One possible explanation for this is the secondary structure differences among HIV-1 subtypes, which may alter the accessibility of the si575 target site.

The approach described here enabled us to select highly effective siRNAs against divergent HIV-1 strains at a high rate. The highly effective siRNAs (>90% inhibition) with maximal conservation (>70%) identified in our study include si521 (poly A site; 94% conservation), si764/770 (Ψ; 88%), si510 (TAR/poly A; 84%), si2075 (ribosomal slip site; 70%), si2329/2330/2333 (protease region; 77%), and si4750/4751/4753 (integrase region; 71–74%). These sites are found mostly in the 5' LTR, protease, and integrase regions (Figure 2). However, the extraordinarily high genetic diversity of HIV-1 obviously prevents us from designing a single siRNA that can nullify all HIV-1 strains currently circulating worldwide (Additional file 7). One possible approach is to combine multiple siRNAs targeting different conserved regions [19,20]. The siRNAs selected and validated in this study have the potential to target >99% of HIV-1 strains by combining only two siRNAs (Additional file 7), and also considered to resist viral mutational escape. Our approach is expected to be highly applicable to therapeutic intervention for other pathogens of public health importance, including HCV, influenza virus, and SARS coronavirus, that are known to show high genetic diversity.

## Competing interests

The author(s) declare that they have no competing interests.

## Authors' contributions

YN performed the computational analyses and the target mRNA cleavage assays, participated in the design of the study, and drafted the manuscript. KN and TO performed the viral replication assays. RU analyzed the data. KU-T participated in the target mRNA cleavage assays, and was involved in critically revising the manuscript. KS and YT supervised the entire study and wrote the manuscript.

## Supplementary Material

Additional file 1 The list of 495 near full-length genome sequences of HIV-1 group M. Click here for file

Additional file 2 The list of published siRNA/shRNAs targeting HIV-1. Click here for file

Additional file 3 The list of highly conserved siRNA targets identified in this study. Click here for file

Additional file 4 The siRNA sequences and their target sites. The sequences of 41 siRNAs and their target sites are shown. The siRNA numbers indicate the nucleotide position in HXB2 (GenBank K03455). The conservation level of each siRNA in HIV-1 group M sequence is depicted in color chart at the rightmost column. BMH (branched multiple hairpin) and LDI (long distance interaction) conformations of the HIV-1 leader RNA and siRNAs targeting them are shown. Click here for file

Additional file 5 Supplementary materials and methods. Click here for file

Additional file 6 Target sites of the 41 siRNAs used in this study. Sequence alignment of the target site from the four HIV-1 infectious molecular clones: pNL4-2 (subtype B); 95MM-yIDU106 (subtype B'); 93IN101 (subtype C); or 93JP-NH1 (CRF01_AE). Click here for file

Additional file 7 Coverage of HIV-1 group M by single siRNA or two siRNAs. (A) Coverage of HIV-1 group M by 41 siRNAs used in this study. (B) Coverage of HIV-1 group M by combining two siRNAs from above. Coverage was calculated by considering only the HIV-1 sequences which contain the corresponding regions. Click here for file
