# Supplementary material for: Optimal design and validation of antiviral siRNA for targeting HIV-1
Source: Retrovirology. 2007 Nov 8;4:80. doi: 10.1186/1742-4690-4-80 (PMC2204037; doi:10.1186/1742-4690-4-80)
Supplement: Additional file 1 — The list of 495 near full-length genome sequences of HIV-1 group M. [file 1742-4690-4-80-S1.pdf]

Accession	Name	Subtype	Country code (ISO3166)	Sampling year
AF193275	97BL006	A1	BY	1997
AF457052	KER2008	A1	KE	2000
AF457053	KER2009	A1	KE	2000
AF457055	KER2012	A1	KE	2000
AF457068	KNH1207	A1	KE	2000
AF457069	KNH1209	A1	KE	2000
AF457070	KNH1211	A1	KE	2000
AF457077	KSM4024	A1	KE	2000
AF457079	KSM4030	A1	KE	2000
AF457080	MSA4069	A1	KE	2000
AF457081	MSA4070	A1	KE	2000
AF457083	MSA4072	A1	KE	2000
AF457084	MSA4076	A1	KE	2000
AF457086	MSA4079	A1	KE	2000
AF457089	NKU3005	A1	KE	2000
AF539405	ML170_1986	A1	KE	1986
AF004885	Q23_17	A1	KE	1994
AF457063	KNH1088	A1	KE	1999
AF457065	KNH1135	A1	KE	1999
AF457075	KSM4021	A1	KE	1999
AF069670	SE7253	A1	SE	1994
AF069671	SE7535	A1	SE	1994
AF069669	SE8538	A1	SE	1995
AF069673	SE8891	A1	SE	1995
AF107771	UGSE8131	A1	SE	1995
AF361872	97TZ02	A1	TZ	1997
AF361873	97TZ03	A1	TZ	1997
AF413987	98UA0116	A1	UA	2000
AB098333	UG029	A1	UG	-
AB098331	UG031	A1	UG	-
M62320	U455	A1	UG	1985
U51190	92UG037	A1	UG	1992
AF484507	98UG57134	A1	UG	1998
AF484508	98UG57135	A1	UG	1998
AF484509	98UG57136	A1	UG	1998
AF484512	98UG57142	A1	UG	1998
AF484478	99UGA07072	A1	UG	1999
AF484493	99UGG03379	A1	UG	1999
AF286240	97CDKFE4	A2	CD	1997
AF286241	97CDKS10	A2	CD	1997
AF286238	97CDKTB48	A2	CD	1997
AF286237	94CY017_41	A2	CY	1994
U23487	contaminant_MANC	B	-	-
AY037269	ARMS008	B	AR	2000
AY037268	ARCH054	B	AR	1998
AY037282	ARMA132	B	AR	1999
AF538302	1181	B	AU	-
AF538303	1182	B	AU	-
AF538304	C24	B	AU	-
AF538305	C42	B	AU	-

Accession	Name	Subtype	Country code (ISO3166)	Sampling year
AF538306	C76	B	AU	-
AF538307	C92	B	AU	-
AF146728	VH	B	AU	-
AF042100	MBC200	B	AU	1986
AF042101	MBC925	B	AU	1987
AF042103	MBCC54	B	AU	1995
AF042104	MBCC98	B	AU	1996
AF042105	MBCD36	B	AU	1996
AY037270	BOL0122	B	BO	1999
AY173956	BZ167	B	BR	1989
AY180905	CNHN24	B	CN	-
U71182	RL42	B	CN	-
U43096	D31	B	DE	1986
U43141	HAN	B	DE	1986
AY173959	EC003	B	EC	1989
AY173960	EC102	B	EC	1989
AF256210	S61K15	B	ES	1989
K03455	HXB2	B	FR	1983
M26727	OYI	B	GA	1988
D10112	CAM1	B	GB	1983
AJ271445	GB8	B	GB	1986
AF224507	WK	B	KR	1997
AB097870	mSTD101	B	MM	1999
U34604	3202A21	B	NL	1986
AY173951	BK132	B	TH	1990
AF086817	TWCYS	B	TW	1994
AY268493	Ac_06	B	US	2001
M17451	RF	B	US	1983
K02007	SF2	B	US	1983
M17449	MNCG	B	US	1984
M38431	NY5CG	B	US	1984
AY352275	SF33	B	US	1984
AF004394	AD87	B	US	1986
U63632	JRFL	B	US	1986
M93258	YU2	B	US	1986
L02317	BC	B	US	1987
AF286365	WR27	B	US	1988
U39362	P896	B	US	1989
AY173952	US1	B	US	1990
AY173953	US2	B	US	1990
AY173954	US3	B	US	1990
AY173955	US4	B	US	1990
U69591	WCIPR	B	US	1990
U21135	WEAU160	B	US	1990
AF069140	DH12_3	B	US	1991
AY308761	1299_d22	B	US	1996
AY308760	1333_d2	B	US	1996
AB078005	ARES2	B	US	1997
AY247251	Ac_06	B	US	1997
U52953	92BR025	C	BR	1992

Accession	Name	Subtype	Country code (ISO3166)	Sampling year
AF286228	98BR004	C	BR	1998
AF443088	00BW07621	C	BW	2000
AF443089	00BW076820	C	BW	2000
AF443090	00BW087421	C	BW	2000
AF443091	00BW147127	C	BW	2000
AF443092	00BW16162	C	BW	2000
AF443093	00BW1686	C	BW	2000
AF443094	00BW17593	C	BW	2000
AF443095	00BW17732	C	BW	2000
AF443096	00BW17835	C	BW	2000
AF443097	00BW17956	C	BW	2000
AF443098	00BW18113	C	BW	2000
AF443099	00BW18595	C	BW	2000
AF443100	00BW18802	C	BW	2000
AF443101	00BW192113	C	BW	2000
AF443102	00BW20361	C	BW	2000
AF443103	00BW20636	C	BW	2000
AF443104	00BW20872	C	BW	2000
AF443105	00BW2127214	C	BW	2000
AF443106	00BW21283	C	BW	2000
AF443107	00BW22767	C	BW	2000
AF443108	00BW38193	C	BW	2000
AF443109	00BW38428	C	BW	2000
AF443110	00BW38713	C	BW	2000
AF443111	00BW3876_9	C	BW	2000
AF443112	00BW3886_8	C	BW	2000
AF443113	00BW3891_6	C	BW	2000
AF443114	00BW3970_2	C	BW	2000
AF443115	00BW5031_1	C	BW	2000
AF110959	96BW01B03	C	BW	1996
AF110962	96BW0402	C	BW	1996
AF110967	96BW0502	C	BW	1996
AF290028	96BW06	C	BW	1996
AF110969	96BW1104	C	BW	1996
AF110972	96BW1210	C	BW	1996
AF110974	96BW15C02	C	BW	1996
AF110976	96BW16B01	C	BW	1996
AF110980	96BW17	C	BW	1996
AF443075	96BWM032	C	BW	1996
AF443074	96BWMO1_5	C	BW	1996
AF443076	98BWMC122	C	BW	1998
AF443077	98BWMC134	C	BW	1998
AF443078	98BWMC14A3	C	BW	1998
AF443079	98BWMO1410	C	BW	1998
AF443080	98BWMO18D5	C	BW	1998
AF443081	98BWMO36A5	C	BW	1998
AF443082	98BWMO37D5	C	BW	1998
AF443083	99BW393212	C	BW	1999
AF443084	99BW46424	C	BW	1999
AF443085	99BW4745	C	BW	1999

Accession	Name	Subtype	Country code (ISO3166)	Sampling year
AF443086	99BW47547	C	BW	1999
AF443087	99BWMC168	C	BW	1999
U46016	ETH2220	C	ET	1986
AF286233	98IS002	C	IL	1998
AB023804	93IN101	C	IN	1993
AF067157	93IN904	C	IN	1993
AF067158	93IN905	C	IN	1993
AF067154	93IN999	C	IN	1993
AF067159	94IN11246	C	IN	1994
AF286223	94IN476	C	IN	1994
AF067155	95IN21068	C	IN	1995
AF286231	98IN012	C	IN	1998
AF286232	98IN022	C	IN	1998
AY049708	01IN565_10	C	IN	1999
AF457054	KER2010	C	KE	2000
AB097871	mIDU101_3	C	MM	1999
AF361874	97TZ04	C	TZ	1997
AF361875	97TZ05	C	TZ	1997
AF286234	98TZ013	C	TZ	1998
AF286235	98TZ017	C	TZ	1998
AY463228	J38MA	C	ZA	2000
AY463223	J54Ma	C	ZA	2001
AY118165	97ZA003	C	ZA	1997
AY118166	97ZA009	C	ZA	1997
AF286227	97ZA012	C	ZA	1997
AY158533	98ZA445	C	ZA	1998
AY158534	98ZA502	C	ZA	1998
AY158535	98ZA528	C	ZA	1998
AY162223	TV001	C	ZA	1998
AY162224	TV002	C	ZA	1998
AY162225	TV012	C	ZA	1998
AF411967	99ZACM9	C	ZA	1999
AY043176	CTSC2	C	ZA	1999
AY043173	DU151	C	ZA	1999
AY043175	DU422	C	ZA	1999
AY043174	DU179	C	ZA	1999
AY463229	1119MB	C	ZA	1999
AY463217	1134MB	C	ZA	2000
AY463218	1176MB	C	ZA	2000
AY463219	1192M3M	C	ZA	2000
AY463220	1195MB	C	ZA	2000
AY463221	1210MB	C	ZA	2000
AY463222	1228MB	C	ZA	2000
AY463224	1162MB	C	ZA	2000
AY463225	1170MB	C	ZA	2000
AY463236	1214MB	C	ZA	2000
AY463226	1217MB	C	ZA	2000
AY463227	1225MB	C	ZA	2000
AY463230	1165MB	C	ZA	2000
AY463231	1168MB	C	ZA	2000

Accession	Name	Subtype	Country code (ISO3166)	Sampling year
AY463232	1171MB	C	ZA	2000
AY463233	1178MB	C	ZA	2000
AY463234	1197MB	C	ZA	2000
AY463235	2004MB	C	ZA	2001
AY463237	2134MB	C	ZA	2001
AF411966	ZASW7	C	ZA	1999
AF286224	96ZM651	C	ZM	1996
AF286225	96ZM751	C	ZM	1996
K03454	ELI	D	CD	1983
M27323	NDK	D	CD	1983
U88822	84ZR085	D	CD	1984
M22639	Z2Z6	D	CD	1985
AY371155	01CM_0009BBY	D	CM	2001
AY371156	01CM_0175BA	D	CM	2001
AY371157	01CM_4412HAL	D	CM	2001
AF133821	MB2059	D	KE	-
AF457090	NKU3006	D	KE	2001
AJ488926	MN011	D	TD	1999
AJ488927	MN012	D	TD	1999
AJ320484	92UG001	D	UG	1992
U88824	94UG114	D	UG	1994
AF484502	98UG57128	D	UG	1998
AF484504	98UG57130	D	UG	1998
AF484505	98UG57131	D	UG	1998
AF484506	98UG57132	D	UG	1998
AF484511	98UG57140	D	UG	1998
AF484514	98UG57143	D	UG	1998
AF484513	98UG57146	D	UG	1998
AF484518	99UGA03349	D	UG	1999
AF484477	99UGA07412	D	UG	1999
AY304496	99UGA08483	D	UG	1999
AF484480	99UGB21875	D	UG	1999
AF484481	99UGB25647	D	UG	1999
AF484483	99UGB32394	D	UG	1999
AF484485	99UGD23550	D	UG	1999
AF484486	99UGD26830	D	UG	1999
AF484487	99UGE08364	D	UG	1999
AF484515	99UGE13613	D	UG	1999
AF484489	99UGE23438	D	UG	1999
AF484519	99UGF03726	D	UG	1999
AF484490	99UGF05734	D	UG	1999
AF484494	99UGG10555	D	UG	1999
AF484495	99UGG35093	D	UG	1999
AF484497	99UGJ27597	D	UG	1999
AF484516	99UGJ32228	D	UG	1998
AF484498	99UGK09259	D	UG	1999
AF484499	99UGK09958	D	UG	1999
AF077336	VI850	F1	BE	1993
AY173957	BZ126	F1	BR	1989
AY173958	BZ163	F1	BR	1989

Accession	Name	Subtype	Country code (ISO3166)	Sampling year
AF005494	93BR020_1	F1	BR	1993
AF075703	FIN9363	F1	FI	1993
AJ249238	MP411	F1	FR	1996
AY371158	02CM_0016BBY	F2	CM	2002
AJ249236	MP255	F2	CM	1995
AJ249237	MP257	F2	CM	1995
AF377956	CM53657	F2	CM	1997
AF084936	DRCBL	G	BE	1996
AY371121	01CM_4049HAN	G	CM	2001
AF423760	X558	G	ES	2000
AF450098	X138	G	ES	1999
AF061641	HH8793_12_1	G	FI	1993
U88826	92NG083	G	NG	1992
AF061642	SE6165	G	SE	1993
AF190127	VI991	H	BE	1993
AF190128	VI997	H	BE	1993
AF005496	056	H	CF	1990
AF082394	SE7887	J	SE	1993
AF082395	SE7022	J	SE	1994
AJ249235	EQTB11C	K	CD	1997
AJ249239	MP535	K	CM	1996
AF197340	90CF11697	01_AE	CF	1990
U51188	90CF402	01_AE	CF	1990
AF197341	90CF4071	01_AE	CF	1990
AY008718	97CNGX_11F	01_AE	CN	1997
AY008714	97CNGX2F	01_AE	CN	1997
AB052995	93JP_NH1	01_AE	JP	1993
AB070352	NH25	01_AE	JP	1993
AF259955	CM235	01_AE	TH	1990
U54771	CM240	01_AE	TH	1990
AF197338	93TH057	01_AE	TH	1993
AF197339	93TH065	01_AE	TH	1993
U51189	93TH253	01_AE	TH	1993
AF164485	93TH9021	01_AE	TH	1993
AB032740	95TNIH022	01_AE	TH	1995
AB032741	95TNIH047	01_AE	TH	1995
AY125894	97TH6_107	01_AE	TH	1997
AY371122	01CM_0002BBY	02_AG	CM	2001
AY371123	01CM_0005BBY	02_AG	CM	2001
AY371124	01CM_0008BBY	02_AG	CM	2001
AY371131	01CM_0074NY	02_AG	CM	2001
AY371137	01CM_0131NY	02_AG	CM	2001
AY371132	01CM_0158ND	02_AG	CM	2001
AY371133	01CM_0191ND	02_AG	CM	2001
AY371134	01CM_0925MO	02_AG	CM	2001
AY371136	01CM_1237NG	02_AG	CM	2001
AY371138	01CM_1475MV	02_AG	CM	2001
AY371142	01CM_4410HAL	02_AG	CM	2001
AY371125	02CM_0013BBY	02_AG	CM	2002
AY371126	02CM_0014BBY	02_AG	CM	2002

Accession	Name	Subtype	Country code (ISO3166)	Sampling year
AY371127	02CM_0015BBY	02_AG	CM	2002
AY371139	02CM_1669LE	02_AG	CM	2002
AY371140	02CM_1677LE	02_AG	CM	2002
AY371146	02CM_1901LE	02_AG	CM	2002
AY371128	02CM_1970LE	02_AG	CM	2002
AY371129	02CM_2162SA	02_AG	CM	2002
AY371130	02CM_2348SA	02_AG	CM	2002
AY371141	02CM_4082STN	02_AG	CM	2002
AJ286133	97CM_MP807	02_AG	CM	1997
AF377954	CM52885	02_AG	CM	1997
AF377955	CM53658	02_AG	CM	1997
AY271690	pBD6_15	02_AG	CM	1999
AY151001	ECU41	02_AG	EC	-
AY151002	ECU42	02_AG	EC	-
AF063223	DJ263	02_AG	FR	1991
AF063224	DJ264	02_AG	FR	1991
AB049811	97GHAG1	02_AG	GH	1997
L39106	IBNG	02_AG	NG	-
AF107770	SE7812	02_AG	SE	1994
AJ251056	MP1211	02_AG	SN	1998
AJ251057	MP1213	02_AG	SN	1998
AF414006	98BY10443	03_AB	BY	2000
AF193276	KAL153	03_AB	RU	1997
AF193277	RU98001	03_AB	RU	1998
AF049337	CY032	04_cpx	CY	1994
AF119820	97PVCH	04_cpx	GR	1991
AF119819	97PVMY	04_cpx	GR	1997
AF193253	VI1310	05_DF	BE	-
AF076998	VI961	05_DF	BE	1993
AY227107	X492	05_DF	ES	1999
AF064699	BFP90	06_cpx	AU	1996
AJ288982	95ML127	06_cpx	ML	1995
AJ245481	95ML84	06_cpx	ML	1995
AJ288981	97SE1078	06_cpx	SN	1997
AF503396	CNGL179	07_BC	CN	-
AF286226	97CN001	07_BC	CN	1997
AX149771	CN54	07_BC	CN	1997
AF286230	98CN009	07_BC	CN	1998
AY008715	97CNGX_6F	08_BC	CN	1997
AY008716	97CNGX_7F	08_BC	CN	1997
AY008717	97CNGX_9F	08_BC	CN	1997
AF286229	98CN006	08_BC	CN	1998
AY093605	96GH2911	09_cpx	GH	1996
AY093603	95SN1795	09_cpx	SN	1995
AY093604	95SN7808	09_cpx	SN	1995
AY093607	99DE4057	09_cpx	US	1999
AF289548	96TZ_BF061	10_CD	TZ	1996
AF289549	96TZ_BF071	10_CD	TZ	1996
AF289550	96TZ_BF110	10_CD	TZ	1996
AY371149	01CM_0186ND	11_cpx	CM	2001

Accession	Name	Subtype	Country code (ISO3166)	Sampling year
AY371150	01CM_4041HAN	11_cpx	CM	2001
AY371151	02CM_2190SA	11_cpx	CM	2002
AY371153	02CM_4118STN	11_cpx	CM	2002
AF492624	1816	11_cpx	CM	1995
AF492623	4496	11_cpx	CM	1996
AJ291718	MP818	11_cpx	CM	1997
AJ291719	MP1298	11_cpx	FR	1999
AJ291720	MP1307	11_cpx	FR	1999
AF179368	GR17	11_cpx	GR	-
AF408629	A32879	12_BF	AR	1997
AF408630	A32989	12_BF	AR	1997
AF385936	ARMA159	12_BF	AR	1999
AF385934	URTR23	12_BF	UY	1999
AF385935	URTR35	12_BF	UY	1999
AY371154	02CM_3226MN	13_cpx	CM	2002
AF460972	1849	13_cpx	CM	1996
AF460974	4164	13_cpx	CM	1996
AF423758	X475	14_BG	ES	2000
AF423759	X477	14_BG	ES	2000
AF450096	X605	14_BG	ES	2000
AF450097	X623	14_BG	ES	2000
AF423756	X397	14_BG	ES	1999
AF423757	X421	14_BG	ES	1999
AF529572	02TH_OUR1331	15_01B	TH	2002
AF529573	02TH_OUR1332	15_01B	TH	2002
AF530576	99TH_R2399	15_01B	TH	1999
AF516184	99TH_MU2079	15_01B	TH	1999
AF286239	97KR004	16_A2D	KR	1997
AF067156	95IN21301	A1C	IN	1995
AF457061	KISII5011	A1C	KE	2000
AF457087	MSA4080	A1C	KE	2000
AF539406	ML170_1995	A1C	KE	1995
AF457064	KNH1097	A1C	KE	1999
U88823	92RW009_06	A1C	RW	1992
AF071474	SE9488	A1C	SE	1996
AF361871	97TZ01	A1C	TZ	1997
AF361876	97TZ06	A1C	TZ	1997
AF361878	97TZ08	A1C	TZ	1997
AF361879	97TZ09	A1C	TZ	1997
AF484491	99UGF25926	A1C	UG	1999
AF484501	99UGK30889	A1C	UG	1999
AF457058	KER2021	A1D	KE	2000
AF457078	KSM4028	A1D	KE	2000
AF457082	MSA4071	A1D	KE	2000
AF457059	KISII5003	A1D	KE	1999
AF457073	KSM4015	A1D	KE	1999
AF075701	SE6954	A1D	SE	1993
AF071473	SE7108	A1D	SE	1994
AF442569	TZBFL0011	A1D	TZ	1996
AF442566	TZBFL0086	A1D	TZ	1997

Accession	Name	Subtype	Country code (ISO3166)	Sampling year
AF442570	TZBFL0088	A1D	TZ	1997
AF484503	98UG57129	A1D	UG	1998
AF484521	98UG57137	A1D	UG	1998
AF484510	98UG57139	A1D	UG	1998
AF484522	98UG57147	A1D	UG	1998
AF484482	99UGB26587	A1D	UG	1999
AF484479	99UGC06443	A1D	UG	1999
AF484517	99UGC38442	A1D	UG	1999
AF484488	99UGE22831	A1D	UG	1999
AF484492	99UGF27390	A1D	UG	1999
AF484496	99UGJ21953	A1D	UG	1999
AF484520	99UGK38855	A1D	UG	1999
AY037267	ARCH003	BF	AR	2000
AF408631	A050	BF	AR	1997
AF408632	A32878	BF	AR	1997
AY037266	ARCH014	BF	AR	1998
AF408626	A025	BF	AR	1999
AF332867	A027	BF	AR	1999
AF408627	A047	BF	AR	1999
AF408628	A063	BF	AR	1999
AY037278	ARMA006	BF	AR	1999
AY037283	ARMA029	BF	AR	1999
AY037275	ARMA036	BF	AR	1999
AY037277	ARMA037	BF	AR	1999
AY037281	ARMA038	BF	AR	1999
AY037280	ARMA097	BF	AR	1999
AY037271	BOL0137	BF	BO	1999
AY037272	URTR17	BF	UY	1999
AB097865	mIDU502	01B	MM	2000
AB097867	mCSW104	01B	MM	1999
AF490973	1269	01B	TH	-
AY167123	CM237	01B	TH	-
AF362994	NP1623	01B	TH	-
AY082968	TH1326	01B	TH	-
AF468970	TH283	01B	TH	-
AF490974	TH9_95	01B	TH	-
AF457088	NKU3004	A1A2D	KE	2000
AF457056	KER2017	A1CD	KE	2000
AY371163	01CM_1152NG	A1U	CM	2001
AF411964	CM4	A1CDGKU	ZA	1999
AF075702	SE8603	A1CD	SE	1995
AJ237565	97NOGIL3	A1DHK	NO	1997
X04415	MAL	A1DK	CD	1985
AY371164	01CM_1404MV	A1F1	CM	2001
AF377959	CM53379	A1F1GHJU	CM	1997
AJ276596	VI1197	A1G	BE	1994
AF377957	CM53392	A1G	CM	1997
AF457062	KNH1043	A1G	KE	1999
U88825	92NG003	A1G	NG	1992
AF076474	VI354	A1GHU	GA	-

Accession	Name	Subtype	Country code (ISO3166)	Sampling year
AF192135	BW2117	A1GJ	BW	1998
U76035	Z321	A1GU	CD	76
AF069672	SE6594	A1U	SE	1993
AF411965	DU178	A2C	ZA	1998
U86780	ZAM184	A2C	ZM	1990
AF457051	KER2003	A2D	KE	1999
AF457072	KSM4001	A2D	KE	1999
AF316544	97CDKP58	A2G	CD	1997
AY371168	01CM_4008HAN	AD	CM	2001
AY371160	02CM_3163MN	AF2	CM	2002
AY371166	01CM_0989MO	AGU	CM	2001
AY371169	02CM_1918LE	AGU	CM	2002
AY371161	01CM_1278NG	AHJU	CM	2001
AY371162	01CM_1296NG	AHJU	CM	2001
AP005206	HH069	BC	CN	2000
AP005207	HH086	BC	CN	2000
AB097873	mIDU103	BC	MM	1999
AB097869	mIDU106	BC	MM	1999
AF005495	93BR029_4	BF1	BR	1993
AF423755	X254	BG	ES	1999
AY074891	00BWMO351	CD	BW	2000
AF457085	MSA4077	CD	KE	2000
AF361877	97TZ07	CD	TZ	1997
AF484500	99UGK10192	CD	UG	1999
AF076475	VI1126	F2KU	BE	1994
AY371159	01CM_0001BBY	01A	CM	2001
AY371165	02CM_1867LE	01A	CM	2002
AY037284	CM53122	01A1	CM	1997
AB097872	mCSW105	01A1	MM	1999
AY371170	01CM_0908MO	01ADF2	CM	2001
AY371167	01CM_0130NY	01AF2U	CM	2001
AB097866	mCSW503	01BC	MM	2000
AB097868	mIDU107	01BC	MM	1999
AY262830	NP1809	01C	TH	-
AJ404325	97DCKTB49	01GHJKU	CD	1997
AY371145	01CM_0190MA	0102A	CM	2001
AF184155	G829	02A	GH	-
AB052867	AG2	02A1	GH	1997
AY371143	02CM_2339SA	02A1U	CM	2002
AJ276595	VI1035	02C	BE	1993
AY371147	02CM_3228MN	02G	CM	2002
AJ293865	B76	06A1	BJ	-
AF286236	83CD003	U	CD	1983
AF457101	90CD121E12	U	CD	1990
AY046058	GR303	U	GR	1999
