# Supplementary material for: Optimal design and validation of antiviral siRNA for targeting HIV-1
Source: Retrovirology. 2007 Nov 8;4:80. doi: 10.1186/1742-4690-4-80 (PMC2204037; doi:10.1186/1742-4690-4-80)
Supplement: Additional file 2 — The list of published siRNA/shRNAs targeting HIV-1. [file 1742-4690-4-80-S2.pdf]

HXB2 coordinate	Target gene	Target site	Conservation (%)	Reference
64	U3, nef	AAGGCTACTTCCCTGATTGGC	34.5	Dave (2004)
67	U3, nef	GCTACTTCCCTGATTGGCAGAACTA	18.6	Yamamoto (2006)
72	U3, nef	TTCCCTGATTGGCAGAACTACACAC	17.9	Yamamoto (2006)
130	U3, nef	GATGGTGCTTCAAGCTAGTAC	28.3	Capodici (2002)
184	U3, nef	AAGGAGAGAACACCAGCTTGT	0.4	Chang (2005)
291	U3, nef	AAGGCCCGAGAGCTGCATCCGG	0.9	Suzuki (2005)
321	U3, nef	AAGACTGCTGACATCGAGCTT	1.1	Suzuki (2005)
348	U3	AAGGGACTTTCCGCTGGGGAC	23.5	Suzuki (2005)
395	U3	AACTGGGGAGTGGCGAGCCCT	0.2	Suzuki (2005)
456	R, TAR	GTCTCTCTGGTTAGACCAGAT	16.2	Yoshinari (2004)
466	R, TAR	TTAGACCAGATCTGAGCCTGG	16.1	Yoshinari (2004)
468	R, TAR	AGACCAGATCTGAGCCTGG	16.1	Jacque (2002)
483	R, TAR	CTGGGAGCTCTCTGGCTAACT	9.5	Yoshinari (2004)
493	R, TAR	TCTGGCTAACTAGGGAACCCA	11.3	Yoshinari (2004)
515	R, poly A	TGCTTAAGCCTCAATAAAGCT	85.3	Yoshinari (2004)
551	R, U5	AAGTAGTGTGTGCCCGTCTGT	65.1	Chang (2005)
626	U5, PBS	TCTCTAGCAGTGGCGCCCG	92.0	Nishitsuji (2006)
634	PBS	GGTGGCGCCCGAACAGGGACCTT	17.0	Han (2004)
635	PBS	GTGGCGCCCGAACAGGGAC	90.1	Das (2004)
636	PBS	TGGCGCCCGAACAGGGACT	54.2	Das (2004)
769	U5, Ψ	GCGGAGGCTAGAAGGAGAG	88.1	Das (2004)
774	U5, Ψ, gag (AUG)	GGCTAGAAGGAGAGAGATG	91.0	Das (2004)
780	U5, gag (AUG)	AAGGAGAGAGATGGGTGCGAGAGCGTC	93.2	ter Brake (2006)
788	U5, gag (AUG)	AGATGGGTGCGAGAGCGTC	92.9	Westerhout (2006b)
896	gag (p17)	GGGCAAGCAGGGAGCTAGAAC	21.5	Capodici (2002)
896	gag (p17)	GGGCAAGCAGGGAGCTAGAACTT	21.5	Han (2004)
1475	gag (p24)	GAGAACCAAGGGGAAGTGACA	44.3	Capodici (2002)
1600	gag (p24)	AATAAAATAGTAAGAATGTA	77.3	ter Brake (2006)
1605	gag (p24)	AATAGTAAGAATGTATAGCCC	75.9	Chang (2005)
1793	gag (p24)	AAGCATTGGGACCAGGAGCGA	0.0	Hu (2002)
1817	gag (p24)	TAGAAGAAATGATGACAGCATG	82.3	ter Brake (2006)
1820	gag (p24)	AAGAAATGATGACAGCATGTC	62.1	Chang (2005)
2060	gag (p7)	AAGATTGTACTGAGAGACAGG	9.3	Novina (2002)
2060	gag (p7)	AAGATTGTACTGAGAGACAGGCT	9.5	Suzuki (2005)
2062	gag (p7)	GATTGTACTGAGAGACAGG	9.3	Song (2003)
2062	gag (p7)	GATTGTACTGAGAGACAGG	9.3	Lee (2005)
2066	gag (p7)	GTACTGAGAGACAGGCTAA	9.1	Pusch (2003)
2077	gag (p7,p1), pol, rebosomal slip site	CAGGCTAATTTTTTAGGGAA	70.6	ter Brake (2006)
2167	gag (p6), pol	GAGGAGAGCTTCAGGTTTGGG	4.2	Lin (2004)
2315	pol (prot)	AGCTCTATTAGATACAGGA	44.5	Paul (2003)
2316	pol (prot)	GCTCTATTAGATACAGGAGCA	47.1	Scherer (2004)
2328	pol (prot)	ACAGGAGCAGATGATACAGT	59.5	ter Brake (2006)
2375	pol (prot)	ATGGAAACCAAAAATGATAGG	81.9	ter Brake (2006)
2559	pol (RT)	CCTATTGAGACTGTACCAG	8.0	Paul (2003)
2586	pol (RT)	AAGCCAGGAATGGATGGCCCA	63.5	Chang (2005)
2748	pol (RT)	GACAGTACTAAATGGAGAATT	0.0	Sabariegos (2005)
2960	pol (RT)	TGAGACACCAGGGATTAGA	22.8	Surabhi (2002)
2961	pol (RT)	GAGACACCAGGGATTAGAT	22.8	Surabhi (2002)
3092	pol (RT)	TCAATACATGGATGATTTGTA	26.5	Huelsmann (2005)
4168	pol (p15)	AAGGAATTGGAGGAAATGAAC	47.2	Chang (2005)

HXB2 coordinate	Target gene	Target site	Conservation (%)	Reference
4173	pol (p15)	ATTGGAGGAAATGAACAAGT	47.6	ter Brake (2006)
4285	pol (int)	GGAGAGCAATGGCTAGTGA	58.7	Nishitsuji (2006)
4537	pol (int)	AATTAGCAGGAAGATGGCCAG	67.0	Chang (2005)
4539	pol (int)	TTAGCAGGAAGATGGCCAGT	67.4	ter Brake (2006)
4650	pol (int)	ATTCCCTACAATCCCCAAAG	50.7	ter Brake (2006)
4776	pol (int), cPPT	CACAATTTTAAAAGAAAAGGGGGGATTGG GGGG	90.7	ter Brake (2006)
4809	pol (int)	TACAGTGCAGGGGAAAGAATA	84.4	ter Brake (2006)
4811	pol (int)	CAGTGCAGGGGAAAGAATA	84.4	Westerhout (2006b)
4884	pol (int), CTS	AAAATTCAAAATTTTCGGGT	77.3	ter Brake (2006)
4888	pol (int), CTS	TTCAAAATTTTCGGGTTTATT	80.6	ter Brake (2006)
4893	pol (int), CTS	AATTTTCGGGTTTATTACAG	78.5	ter Brake (2006)
4953	pol (int)	CTCTGGAAAGGTGAAGGGGCAGTAGTAAT	84.0	ter Brake (2006)
4960	pol (int)	AAGGTGAAGGGGCAGTAGTAA	69.8	Chang (2005)
4966	pol (int)	AAGGGGCAGTAGTAATAGAAG	0.0	Hu (2002)
5040	pol (int), vif	TATGGAAAACAGATGGCAGGTG	90.7	ter Brake (2006)
5138	vif	GGAAAGCTAAGGACTGGTT	0.2	Jacque (2002)
5138	vif	GGAAAGCTAAGGACTGGTT	0.2	Joshi (2005)
5195	vif	GTTCAGAAGTACACATCCC	60.8	Lee (2005)
5323	vif	AGCACACAAGTAGACCCTGA	24.4	Jacque (2002)
5323	vif	AGCACACAAGTAGACCCTG	24.4	Jacque (2002)
5323	vif	AGCACACAAGTAGACCCTG	24.4	Nishitsuji (2004)
5481	vif	CTTGGCACTAGCAGCATTA	5.6	Jacque (2002)
5852	tat	CTAGAGCCCTGGAAGCATC	7.8	Surabhi (2002)
5864	tat	AAGCATCCAGGAAGTCAGCCT	10.3	Dave (2004)
5887	tat	AACTGCTTGTACCAATTGCTA	1.8	Coburn (2002)
5889	tat	CTGCTTGTACCAATTGCTATT	5.0	Boden (2003a,b)
5889	tat	CTGCTTGTACCAATTGCTA	3.2	Boden (2004b)
5889	tat	CTGCTTGTACCAATTGCTATT	5.0	Huelsmann (2005)
5915	tat	AAGTGTTGCTTTCATTGCCAAG	5.4	Lee (2003)
5954	tat, rev	GCCTTAGGCATCTCCTATG	1.2	Das (2004)
5966	tat, rev	TCCTATGGCAGGAAGAAGCGGAG	81.5	ter Brake (2006)
5967	tat, rev	CCTATGGCAGGAAGAAGCG	56.8	Das (2004)
5969	tat, rev	TATGGCAGGAAGAAGCGGA	79.9	Surabhi (2002)
5980	tat, rev	GAAGCGGAGACAGCGACGA	45.5	Surabhi (2002)
5983	tat, rev	GCGGAGACAGCGACGAAGAGC	7.4	Lee (2002)
5983	tat, rev	GCGGAGACAGCGACGAAGAGC	7.4	Li (2003)
5983	tat, rev	GCGGAGACAGCGACGAAGAGC	7.4	Scherer (2004)
5983	tat, rev	GCGGAGACAGCGACGAAGAGC	7.4	Joshi (2005)
5983	tat, rev	GCGGAGACAGCGACGAAGAGC	7.4	Li (2005)
5994	tat, rev	GACGAAGAGCTCATCAGAACA	0.2	Boden (2004a)
6205	vpu	AAGAGCAGAAGACAGTGGCAA	53.6	Chang (2005)
6213	vpu, env	AAGACAGTGGCAATGAGAGTG	52.2	Chang (2005)
7152	env	AAGAGGGGACCAGGGAGAGCAC	0.0	Park (2003)
7368	env	AAGTTCTACTGTAATTCAACAC	0.0	Park (2003)
7384	env	CAACACAACTGTTTAATAGTA	4.0	Scherer (2004)
7460	env	CACAATCACACTCCCATGCAG	1.0	Scherer (2004)
7464	env	AAGACACTCCCATGCAGAATAC	0.0	Park (2003)
7497	env	AAGATGTGGCAGGAAGTAGGAC	0.2	Park (2003)
7635	env	GGAGGAGGCGATATGAGGGAC	0.4	Scherer (2004)
7798	env	CAGCAGGAAGCACTATGGGCGC	75.8	ter Brake (2006)

HXB2 coordinate	Target gene	Target site	Conservation (%)	Reference
7858	env	TGTCTGATATAGTGCAGCAGC	0.0	Scherer (2004)
7916	env	TCTGTTGCAACTCACAGTCTG	24.1	Scherer (2004)
7924	env	AACTCACAGTCTGGGGCATCA	11.9	Dave (2004)
8425	tat, rev, env	AAGAAGAAGGTGGAGAGAGAG	7.1	Dave (2004)
8472	rev, env	AACGGATCCTTGGCACTTATC	0.4	Coburn (2002)
8483	rev, env	GGCACTTATCTGGGACGAT	0.4	Lee (2005)
8509	rev, env	GCCTGTGCCTCTTCAGCTACC	40.6	Lee (2002)
8509	rev, env	GCCTGTGCCTCTTCAGCTACC	40.6	Banerjea (2003)
8509	rev, env	GCCTGTGCCTCTTCAGCTACC	40.6	Li (2003)
8509	rev, env	GCCTGTGCCTCTTCAGCTACC	40.6	Scherer (2004)
8509	rev, env	GCCTGTGCCTCTTCAGCTACC	40.6	Unwalla (2004)
8509	rev, env	GCCTGTGCCTCTTCAGCTACC	40.6	Joshi (2005)
8509	rev, env	GCCTGTGCCTCTTCAGCTACC	40.6	Unwalla (2006)
8558	rev, env	AACGAGGATTGTGGAACTTCT	8.7	Dave (2004)
8759	env	AAGAATAAGACAGGGCTTGGA	9.5	Dave (2004)
8762	env	AATAAGACAGGGCTTGGAAAG	10.9	Dave (2004)
8817	nef	AAGTAGTGTGATTGGATGGCC	0.4	Chang (2005)
8844	nef	AAGGGAAAGAATGAGACGAGC	6.3	Dave (2004)
8914	nef	CAUGGAGCAAUCACAAGUATT	0.0	Sabariegos (2005)
8960	nef	GTGCCTGGCTAGAAGCACA	11.6	Jacque (2002)
8960	nef	GTGCCTGGCTAGAAGCACA	11.6	Das (2004)
8960	nef	GTGCCTGGCTAGAAGCACA	11.6	Westerhout (2005)
8960	nef	GTGCCTGGCTAGAAGCACA	11.6	Westerhout (2006a,b)
8999	nef	TTCCAGTCACACCTCAGGTAC	1.6	Scherer (2004)
9063	nef, U3	CTTTTTAAAAGAAAAGGGGGGACTGGA	90.7	ter Brake (2006)
9069	nef, U3	AAAAGAAAAGGGGGGACTGGA	90.7	Dave (2004)
9078	nef, U3	GGGGGGACTGGAAGGGCTA	14.1	Das (2004)
9081	nef, U3	GGGACTGGAAGGGCTAATT	10.6	Westerhout (2006b)
9083	nef, U3	GACTGGAAGGGCTAATTCA	2.6	Nishitsuji (2006)

* For the target sequence longer than 21 nt, conservation was calculated for each possible 21 nt subsequence, and the highest value is shown. Target sequence shorter than 21 nt was extended to 21 nt for calculating their conservations.

Additional file 2. (Naito, Y. *et al.*)

References

1. Banerjea A, Li MJ, Bauer G, Remling L, Lee NS, Rossi J, Akkina R: **Inhibition of HIV-1 by lentiviral vector-transduced siRNAs in T lymphocytes differentiated in SCID-hu mice and CD34^{+} progenitor cell-derived macrophages.** *Mol Ther* 2003, **8:**62-71.
2. Boden D, Pusch O, Lee F, Tucker L, Shank PR, Ramratnam B: **Promoter choice affects the potency of HIV-1 specific RNA interference.** *Nucleic Acids Res* 2003a, **31:**5033-5038.
3. Boden D, Pusch O, Lee F, Tucker L, Ramratnam B: **Human immunodeficiency virus type 1 escape from RNA interference.** *J Virol* 2003b, **77:**11531-11535.
4. Boden D, Pusch O, Silbermann R, Lee F, Tucker L, Ramratnam B: **Enhanced gene silencing of HIV-1 specific siRNA using microRNA designed hairpins.** *Nucleic Acids Res* 2004a, **32:**1154-1158.
5. Boden D, Pusch O, Lee F, Tucker L, Ramratnam B: **Efficient gene transfer of HIV-1-specific short hairpin RNA into human lymphocytic cells using recombinant adeno-associated virus vectors.** *Mol Ther* 2004b, **9:**396-402.
6. Capodici J, Karikó K, Weissman D: **Inhibition of HIV-1 infection by small interfering RNA-mediated RNA interference.** *J Immunol* 2002, **169:**5196-5201.
7. Chang LJ, Liu X, He J: **Lentiviral siRNAs targeting multiple highly conserved RNA sequences of human immunodeficiency virus type 1.** *Gene Ther* 2005, **12:**1133-1144.
8. Coburn GA, Cullen BR: **Potent and specific inhibition of human immunodeficiency virus type 1 replication by RNA interference.** *J Virol* 2002, **76:**9225-9231.
9. Das AT, Brummelkamp TR, Westerhout EM, Vink M, Madiredjo M, Bernards R, Berkhout B: **Human immunodeficiency virus type 1 escapes from RNA interference-mediated inhibition.** *J Virol* 2004, **78:**2601-2605.
10. Dave RS, Pomerantz RJ: **Antiviral effects of human immunodeficiency virus type 1-specific small interfering RNAs against targets conserved in select neurotropic viral strains.** *J Virol* 2004, **78:**13687-13696.
11. Han W, Wind-Rotolo M, Kirkman RL, Morrow CD: **Inhibition of human immunodeficiency virus type 1 replication by siRNA targeted to the highly conserved primer binding site.** *Virology* 2004, **330:**221-232.
12. Hu WY, Myers CP, Kilzer JM, Pfaff SL, Bushman FD: **Inhibition of retroviral pathogenesis by RNA interference.** *Curr Biol* 2002, **12:**1301-1311.
13. Huelsmann PM, Rauch P, Allers K, John MJ, Metzner KJ: **Inhibition of drug-resistant HIV-1 by RNA interference.** *Antiviral Res* 2006, **69:**1-8.
14. Jacque JM, Triques K, Stevenson M: **Modulation of HIV-1 replication by RNA interference.** *Nature* 2002, **418:**435-438.

15. Joshi PJ, North TW, Prasad VR: **Aptamers directed to HIV-1 reverse transcriptase display greater efficacy over small hairpin RNAs targeted to viral RNA in blocking HIV-1 replication.** *Mol Ther* 2005, **11:**677-686.
16. Lee MTM, Coburn GA, McClure MO, Cullen BR: **Inhibition of human immunodeficiency virus type 1 replication in primary macrophages by using Tat- or CCR5-specific small interfering RNAs expressed from a lentivirus vector.** *J Virol* 2003, **77:**11964-11972.
17. Lee NS, Dohjima T, Bauer G, Li H, Li MJ, Ehsani A, Salvaterra P, Rossi J: **Expression of small interfering RNAs targeted against HIV-1 *rev* transcripts in human cells.** *Nat Biotechnol* 2002, **20:**500-505.
18. Lee SK, Dykxhoorn DM, Kumar P, Ranjbar S, Song E, Maliszewski LE, François-Bongarçon V, Goldfeld A, Swamy NM, Lieberman J, Shankar P: **Lentiviral delivery of short hairpin RNAs protects CD4 T cells from multiple clades and primary isolates of HIV.** *Blood* 2005, **106:**818-826.
19. Li MJ, Bauer G, Michienzi A, Yee JK, Lee NS, Kim J, Li S, Castanotto D, Zaia J, Rossi JJ: **Inhibition of HIV-1 infection by lentiviral vectors expressing Pol III-promoted anti-HIV RNAs.** *Mol Ther* 2003, **8:**196-206.
20. Li MJ, Kim J, Li S, Zaia J, Yee JK, Anderson J, Akkina R, Rossi JJ: **Long-term inhibition of HIV-1 infection in primary hematopoietic cells by lentiviral vector delivery of a triple combination of anti-HIV shRNA, anti-CCR5 ribozyme, and a nucleolar-localizing TAR decoy.** *Mol Ther* 2005, **12:**900-909.
21. Lin SL, Ying SY: **Combinational therapy for potential HIV-1 eradication and vaccination.** *Int J Oncol* 2004, **24:**81-88.
22. Nishitsuji H, Ikeda T, Miyoshi H, Ohashi T, Kannagi M, Masuda T: **Expression of small hairpin RNA by lentivirus-based vector confers efficient and stable gene-suppression of HIV-1 on human cells including primary non-dividing cells.** *Microbes Infect* 2004, **6:**76-85.
23. Nishitsuji H, Kohara M, Kannagi M, Masuda T: **Effective suppression of human immunodeficiency virus type 1 through a combination of short- or long-hairpin RNAs targeting essential sequences for retroviral integration.** *J Virol* 2006, **80:**7658-7666.
24. Novina CD, Murray MF, Dykxhoorn DM, Beresford PJ, Riess J, Lee SK, Collman RG, Lieberman J, Shankar P, Sharp PA: **siRNA-directed inhibition of HIV-1 infection.** *Nat Med* 2002, **8:**681-686.
25. Park WS, Hayafune M, Miyano-Kurosaki N, Takaku H: **Specific HIV-1 env gene silencing by small interfering RNAs in human peripheral blood mononuclear cells.** *Gene Ther* 2003, **10:**2046-2050.
26. Paul CP, Good PD, Li SXL, Kleihauer A, Rossi JJ, Engelke DR: **Localized expression of small RNA inhibitors in human cells.** *Mol Ther* 2003, **7:**237-247.

27. Pusch O, Boden D, Silbermann R, Lee F, Tucker L, Ramratnam B: **Nucleotide sequence homology requirements of HIV-1-specific short hairpin RNA.** *Nucleic Acids Res* 2003, **31:**6444-6449.
28. Sabariegos R, Giménez-Barcons M, Tàpia N, Clotet B, Martínez MA: **Sequence homology required by human immunodeficiency virus type 1 to escape from short interfering RNAs.** *J Virol* 2006, **80:**571-577.
29. Scherer LJ, Yildiz Y, Kim J, Cagnon L, Heale B, Rossi JJ: **Rapid assessment of anti-HIV siRNA efficacy using PCR-derived Pol III shRNA cassettes.** *Mol Ther* 2004, **10:**597-603.
30. Song E, Lee SK, Dykxhoorn DM, Novina C, Zhang D, Crawford K, Cerny J, Sharp PA, Lieberman J, Manjunath N, Shankar P: **Sustained small interfering RNA-mediated human immunodeficiency virus type 1 inhibition in primary macrophages.** *J Virol* 2003, **77:**7174-7181.
31. Surabhi RM, Gaynor RB: **RNA interference directed against viral and cellular targets inhibits human immunodeficiency virus type 1 replication.** *J Virol* 2002, **76:**12963-12973.
32. Suzuki K, Shijuuku T, Fukamachi T, Zaunders J, Guillemin G, Cooper D, Kelleher A: **Prolonged transcriptional silencing and CpG methylation induced by siRNAs targeted to the HIV-1 promoter region.** *J RNAi Gene Silencing* 2005, **1:**66-78.
33. ter Brake O, Konstantinova P, Ceylan M, Berkhout B: **Silencing of HIV-1 with RNA interference: a multiple shRNA approach.** *Mol Ther* 2006, **14:**883-892.
34. Unwalla HJ, Li MJ, Kim JD, Li HT, Ehsani A, Alluin J, Rossi JJ: **Negative feedback inhibition of HIV-1 by TAT-inducible expression of siRNA.** *Nat Biotechnol* 2004, **22:**1573-1578.
35. Unwalla HJ, Li HT, Bahner I, Li MJ, Kohn D, Rossi JJ: **Novel Pol II fusion promoter directs human immunodeficiency virus type 1-inducible coexpression of a short hairpin RNA and protein.** *J Virol* 2006, **80:**1863-1873.
36. Westerhout EM, Ooms M, Vink M, Das AT, Berkhout B: **HIV-1 can escape from RNA interference by evolving an alternative structure in its RNA genome.** *Nucleic Acids Res* 2005, **33:**796-804.
37. Westerhout EM, Vink M, Haasnoot PCJ, Das AT, Berkhout B: **A conditionally replicating HIV-based vector that stably expresses an antiviral shRNA against HIV-1 replication.** *Mol Ther* 2006a, **14:**268-275.
38. Westerhout EM, ter Brake O, Berkhout B: **The virion-associated incoming HIV-1 RNA genome is not targeted by RNA interference.** *Retrovirology* 2006b, **3:**57.
39. Yamamoto T, Miyoshi H, Yamamoto N, Yamamoto N, Inoue J, Tsunetsugu-Yokota Y: **Lentivirus vectors expressing short hairpin RNAs against the *U3*-overlapping region of HIV *nef* inhibit HIV replication and infectivity in primary macrophages.** *Blood* 2006, **108:**3305-3312.
40. Yoshinari K, Miyagishi M, Taira K: **Effects on RNAi of the tight structure, sequence and position of the targeted region.** *Nucleic Acids Res* 2004, **32:**691-699.
