# Supplementary material for: Optimal design and validation of antiviral siRNA for targeting HIV-1
Source: Retrovirology. 2007 Nov 8;4:80. doi: 10.1186/1742-4690-4-80 (PMC2204037; doi:10.1186/1742-4690-4-80)
Supplement: Additional file 3 — The list of highly conserved siRNA targets identified in this study. [file 1742-4690-4-80-S3.pdf]

HXB2 coordinate	Target gene	Target site (21 bp)	Conservation (%)	siRNA efficacy prediction Ui-Tei	Reynolds	Amarzguioui
412	U3, TATA	CCCTCAGATGCTGCATATAAG	74.6			
413	U3, TATA	CCTCAGATGCTGCATATAAGC	75.1			
414	U3, TATA	CTCAGATGCTGCATATAAGCA	74.6	yes		
415	U3, TATA	TCAGATGCTGCATATAAGCAG	74.6			
416	U3, TATA	CAGATGCTGCATATAAGCAGC	75.8			
505	R, TAR	GGGAACCCACTGCTTAAGCCT	77.8	yes		
506	R, TAR	GGAACCCACTGCTTAAGCCTC	81.3			
507	R, TAR, poly A	GAACCCACTGCTTAAGCCTCA	84.9			
508	R, TAR, poly A	AACCCACTGCTTAAGCCTCAA	85.3			yes
509	R, TAR, poly A	ACCCACTGCTTAAGCCTCAAT	84.9	yes	yes	yes
510	R, TAR, poly A	CCCACTGCTTAAGCCTCAATA	84.9	yes	yes	yes
511	R, TAR, poly A	CCACTGCTTAAGCCTCAATAA	85.2		yes	
512	R, poly A	CACTGCTTAAGCCTCAATAAA	85.4	yes	yes	yes
513	R, poly A	ACTGCTTAAGCCTCAATAAAG	84.7			
514	R, poly A	CTGCTTAAGCCTCAATAAAGC	85.3			
515	R, poly A	TGCTTAAGCCTCAATAAAGCT	85.3	yes		
516	R, poly A	GCTTAAGCCTCAATAAAGCTT	85.7			
517	R, poly A	CTTAAGCCTCAATAAAGCTTG	85.7			
518	R, poly A	TTAAGCCTCAATAAAGCTTGC	86.7			
519	R, poly A	TAAGCCTCAATAAAGCTTGCC	86.6			
520	R, poly A	AAGCCTCAATAAAGCTTGCCT	93.9			yes
521	R, poly A	AGCCTCAATAAAGCTTGCCTT	94.2	yes		yes
522	R, poly A	GCCTCAATAAAGCTTGCCTTG	95.0			
523	R, poly A	CCTCAATAAAGCTTGCCTTGA	94.2			
524	R, poly A	CTCAATAAAGCTTGCCTTGAG	94.6			
525	R, poly A	TCAATAAAGCTTGCCTTGAGT	87.5			
526	R, poly A	CAATAAAGCTTGCCTTGAGTG	87.5			
527	R, poly A	AATAAAGCTTGCCTTGAGTGC	85.5			
528	R, poly A	ATAAAGCTTGCCTTGAGTGCT	79.5			
552	U5	AGTAGTGTGTGCCCGTCTGTT	70.3			
576	U5, PAS	TGACTCTGGTAACTAGAGATC	71.5			
577	U5, PAS	GACTCTGGTAACTAGAGATCC	81.3			
578	U5, PAS	ACTCTGGTAACTAGAGATCCC	81.9			
579	U5, PAS	CTCTGGTAACTAGAGATCCCT	87.1			yes
580	U5, PAS	TCTGGTAACTAGAGATCCCTC	87.1			
581	U5, PAS	CTGGTAACTAGAGATCCCTCA	87.7			yes
582	U5, PAS	TGGTAACTAGAGATCCCTCAG	88.2			
583	U5, PAS	GGTAACTAGAGATCCCTCAGA	88.2		yes	
584	U5, PAS	GTAACTAGAGATCCCTCAGAC	87.7			
585	U5	TAACTAGAGATCCCTCAGACC	83.7			
622	U5, PBS	AAAATCTCTAGCAGTGGCGCC	90.2			
623	U5, PBS	AAATCTCTAGCAGTGGCGCCC	93.1			
624	U5, PBS	AATCTCTAGCAGTGGCGCCCG	92.0			
625	U5, PBS	ATCTCTAGCAGTGGCGCCCGA	92.1			
626	U5, PBS	TCTCTAGCAGTGGCGCCCGAA	93.3			
627	U5, PBS	CTCTAGCAGTGGCGCCCGAAC	92.8			
628	U5, PBS	TCTAGCAGTGGCGCCCGAACA	92.4			
629	U5, PBS	CTAGCAGTGGCGCCCGAACAG	92.4			
630	U5, PBS	TAGCAGTGGCGCCCGAACAGG	93.8			
631	U5, PBS	AGCAGTGGCGCCCGAACAGGG	93.3			

HXB2 coordinate	Target gene	Target site (21 bp)	Conservation (%)	siRNA efficacy prediction Ui-Tei	Reynolds	Amarzguioui
632	U5, PBS	GCAGTGGCGCCCGAACAGGGA	90.0			
633	U5, PBS	CAGTGGCGCCCGAACAGGGAC	90.1			
682	U5	TCTCTCGACGCAGGACTCGGC	85.2			
683	U5	CTCTCGACGCAGGACTCGGCT	88.1			
684	U5	TCTCGACGCAGGACTCGGCTT	88.5			
685	U5	CTCGACGCAGGACTCGGCTTG	88.5			
686	U5	TCGACGCAGGACTCGGCTTGC	88.2			
687	U5	CGACGCAGGACTCGGCTTGCT	88.5			
688	U5	GACGCAGGACTCGGCTTGCTG	90.0			
689	U5	ACGCAGGACTCGGCTTGCTGA	88.2	yes		yes
761	U5, Ψ	TTTGACTAGCGGAGGCTAGAA	85.2		yes	
762	U5, Ψ	TTGACTAGCGGAGGCTAGAAG	86.9			
763	U5, Ψ	TGACTAGCGGAGGCTAGAAGG	87.6			
764	U5, Ψ	GACTAGCGGAGGCTAGAAGGA	88.0	yes		
765	U5, Ψ	ACTAGCGGAGGCTAGAAGGAG	88.0			
766	U5, Ψ	CTAGCGGAGGCTAGAAGGAGA	88.4			yes
767	U5, Ψ	TAGCGGAGGCTAGAAGGAGAG	88.1			
768	U5, Ψ	AGCGGAGGCTAGAAGGAGAGA	87.8			yes
769	U5, Ψ	GCGGAGGCTAGAAGGAGAGAG	88.3			
770	U5, Ψ, gag (AUG)	CGGAGGCTAGAAGGAGAGAGA	88.6	yes		yes
771	U5, Ψ, gag (AUG)	GGAGGCTAGAAGGAGAGAGAT	88.3			yes
772	U5, Ψ, gag (AUG)	GAGGCTAGAAGGAGAGAGATG	91.0			
773	U5, Ψ, gag (AUG)	AGGCTAGAAGGAGAGAGATGG	91.4			
774	U5, Ψ, gag (AUG)	GGCTAGAAGGAGAGAGATGGG	91.4			
775	U5, Ψ, gag (AUG)	GCTAGAAGGAGAGAGATGGGT	91.1			
776	U5, Ψ, gag (AUG)	CTAGAAGGAGAGAGATGGGTG	90.4			
777	U5, Ψ, gag (AUG)	TAGAAGGAGAGAGATGGGTGC	90.4			
778	U5, Ψ, gag (AUG)	AGAAGGAGAGAGATGGGTGCG	90.1			
779	U5, Ψ, gag (AUG)	GAAGGAGAGAGATGGGTGCGA	90.4			
780	U5, gag (AUG)	AAGGAGAGAGATGGGTGCGAG	92.1			
781	U5, gag (AUG)	AGGAGAGAGATGGGTGCGAGA	92.1			yes
782	U5, gag (AUG)	GGAGAGAGATGGGTGCGAGAG	92.1			
783	U5, gag (AUG)	GAGAGAGATGGGTGCGAGAGC	93.2			
784	U5, gag (AUG)	AGAGAGATGGGTGCGAGAGCG	92.5			
785	U5, gag (AUG)	GAGAGATGGGTGCGAGAGCGT	93.2			
786	U5, gag (AUG)	AGAGATGGGTGCGAGAGCGTC	92.9			
787	U5, gag (AUG)	GAGATGGGTGCGAGAGCGTCA	89.8			
1597	gag (p24)	TTAAATAAAATAGTAAGAATG	72.4			
1598	gag (p24)	TAAATAAAATAGTAAGAATGT	77.3			
1599	gag (p24)	AAATAAAATAGTAAGAATGTA	76.9		yes	
1600	gag (p24)	AATAAAATAGTAAGAATGTAT	77.7			
1601	gag (p24)	ATAAAATAGTAAGAATGTATA	77.7		yes	
1602	gag (p24)	TAAAATAGTAAGAATGTATAG	77.5			
1603	gag (p24)	AAAATAGTAAGAATGTATAGC	75.9			
1604	gag (p24)	AAATAGTAAGAATGTATAGCC	75.9			
1605	gag (p24)	AATAGTAAGAATGTATAGCCC	75.9			
1606	gag (p24)	ATAGTAAGAATGTATAGCCCT	75.9			
1817	gag (p24)	TAGAAGAAATGATGACAGCAT	82.3	yes		yes
1818	gag (p24)	AGAAGAAATGATGACAGCATG	82.3			
2075	gag (p7, p1), pol, ribosomal slip site	GACAGGCTAATTTTTTAGGGA	70.6	yes		

HXB2 coordinate	Target gene	Target site (21 bp)	Conservation (%)	siRNA efficacy prediction Ui-Tei	Reynolds	Amarzguioui
2077	gag (p7, p1), pol, ribosomal slip site	CAGGCTAATTTTTTAGGGAAA	70.0	yes		yes
2251	gag (p6), pol (prot)	TCCCTCAAATCACTCTTTGGC	79.9			
2252	gag (p6), pol (prot)	CCCTCAAATCACTCTTTGGCA	81.9	yes		yes
2328	pol (prot)	ACAGGAGCAGATGATACAGTA	81.3		yes	
2329	pol (prot)	CAGGAGCAGATGATACAGTAT	77.7	yes	yes	yes
2330	pol (prot)	AGGAGCAGATGATACAGTATT	77.9	yes		yes
2331	pol (prot)	GGAGCAGATGATACAGTATTA	79.1		yes	yes
2332	pol (prot)	GAGCAGATGATACAGTATTAG	78.5			
2333	pol (prot)	AGCAGATGATACAGTATTAGA	77.3	yes		yes
2334	pol (prot)	GCAGATGATACAGTATTAGAA	79.7		yes	
2335	pol (prot)	CAGATGATACAGTATTAGAAG	78.3			
2336	pol (prot)	AGATGATACAGTATTAGAAGA	77.7			
2375	pol (prot)	ATGGAAACCAAAAATGATAGG	81.9			
2487	pol (prot)	CCTACACCTGTCAACATAATT	72.0			
2488	pol (prot)	CTACACCTGTCAACATAATTG	72.2			
2489	pol (prot)	TACACCTGTCAACATAATTGG	72.2			
2490	pol (prot)	ACACCTGTCAACATAATTGGA	73.0			
2611	pol (RT)	TTAAACAATGGCCATTGACAG	70.0			
2613	pol (RT)	AAACAATGGCCATTGACAGAA	73.0			
2614	pol (RT)	AACAATGGCCATTGACAGAAG	73.0			
2615	pol (RT)	ACAATGGCCATTGACAGAAGA	73.6		yes	
2696	pol (RT)	AATTGGGCCTGAAAATCCATA	74.8			
3302	pol (RT)	CTGGACTGTCAATGATATACA	70.2	yes	yes	yes
4173	pol (p15)	ATTGGAGGAAATGAACAAGTA	76.7		yes	
4174	pol (p15)	TTGGAGGAAATGAACAAGTAG	76.5			
4175	pol (p15)	TGGAGGAAATGAACAAGTAGA	76.5	yes		yes
4176	pol (p15)	GGAGGAAATGAACAAGTAGAT	71.1			yes
4177	pol (p15)	GAGGAAATGAACAAGTAGATA	71.1	yes		yes
4178	pol (p15)	AGGAAATGAACAAGTAGATAA	70.5	yes	yes	yes
4179	pol (p15)	GGAAATGAACAAGTAGATAAA	70.7		yes	
4182	pol (p15)	AATGAACAAGTAGATAAATTA	71.7		yes	
4183	pol (p15)	ATGAACAAGTAGATAAATTAG	71.9			
4184	pol (p15)	TGAACAAGTAGATAAATTAGT	71.9			
4749	pol (int)	ACAGCAGTACAAATGGCAGTA	74.9			
4750	pol (int)	CAGCAGTACAAATGGCAGTAT	74.1	yes		yes
4751	pol (int)	AGCAGTACAAATGGCAGTATT	74.1	yes	yes	yes
4752	pol (int)	GCAGTACAAATGGCAGTATTC	71.5			
4753	pol (int)	CAGTACAAATGGCAGTATTCA	71.5	yes	yes	yes
4754	pol (int)	AGTACAAATGGCAGTATTCAT	71.5			
4776	pol (int), cPPT	CACAATTTTAAAAGAAAAGGG	89.8			
4777	pol (int), cPPT	ACAATTTTAAAAGAAAAGGGG	89.8			
4778	pol (int), cPPT	CAATTTTAAAAGAAAAGGGGG	89.8			
4779	pol (int), cPPT	AATTTTAAAAGAAAAGGGGGG	90.5			
4780	pol (int), cPPT	ATTTTAAAAGAAAAGGGGGGA	90.5			
4781	pol (int), cPPT	TTTTAAAAGAAAAGGGGGGAT	90.5			
4782	pol (int), cPPT	TTTAAAAGAAAAGGGGGGATT	90.7			
4783	pol (int), cPPT	TTAAAAGAAAAGGGGGGATTG	90.7			
4784	pol (int), cPPT	TAAAAGAAAAGGGGGGATTGG	90.7			
4785	pol (int), cPPT	AAAAGAAAAGGGGGGATTGGG	90.3			
4786	pol (int), cPPT	AAAGAAAAGGGGGGATTGGGG	89.4			

HXB2 coordinate	Target gene	Target site (21 bp)	Conservation (%)	siRNA efficacy prediction Ui-Tei	Reynolds	Amarzguioui
4787	pol (int), cPPT	AAGAAAAGGGGGGATTGGGGG	86.6			
4788	pol (int), cPPT	AGAAAAGGGGGGATTGGGGGG	78.1			
4789	pol (int), cPPT	GAAAAGGGGGGATTGGGGGGT	78.1			
4790	pol (int), cPPT	AAAAGGGGGGATTGGGGGGTA	78.1			
4791	pol (int), cPPT	AAAGGGGGGATTGGGGGGTAC	76.9			
4792	pol (int), cPPT	AAGGGGGGATTGGGGGGTACA	76.7			yes
4793	pol (int), cPPT	AGGGGGGATTGGGGGGTACAG	80.2			
4794	pol (int), cPPT	GGGGGGATTGGGGGGTACAGT	80.4	yes		yes
4795	pol (int), cPPT	GGGGGATTGGGGGGTACAGTG	80.4			
4796	pol (int), cPPT	GGGGATTGGGGGGTACAGTGC	80.0			
4797	pol (int), cPPT	GGGATTGGGGGGTACAGTGCA	79.3			
4798	pol (int), cPPT	GGATTGGGGGGTACAGTGCAG	78.9			
4799	pol (int), cPPT	GATTGGGGGGTACAGTGCAGG	79.1			
4800	pol (int), cPPT	ATTGGGGGGTACAGTGCAGGG	76.1			
4801	pol (int)	TTGGGGGGTACAGTGCAGGGG	75.9			
4802	pol (int)	TGGGGGGTACAGTGCAGGGGA	75.5			yes
4803	pol (int)	GGGGGGTACAGTGCAGGGGAA	73.5			yes
4804	pol (int)	GGGGGTACAGTGCAGGGGAAA	72.9			yes
4805	pol (int)	GGGGTACAGTGCAGGGGAAAG	73.1			
4806	pol (int)	GGGTACAGTGCAGGGGAAAGA	72.3	yes	yes	
4807	pol (int)	GGTACAGTGCAGGGGAAAGAA	72.9			
4808	pol (int)	GTACAGTGCAGGGGAAAGAAT	74.5		yes	
4809	pol (int)	TACAGTGCAGGGGAAAGAATA	84.4	yes	yes	yes
4810	pol (int)	ACAGTGCAGGGGAAAGAATAA	71.5		yes	yes
4811	pol (int)	CAGTGCAGGGGAAAGAATAAT	71.5	yes		yes
4812	pol (int)	AGTGCAGGGGAAAGAATAATA	73.7		yes	
4813	pol (int)	GTGCAGGGGAAAGAATAATAG	73.9			
4814	pol (int)	TGCAGGGGAAAGAATAATAGA	74.1	yes	yes	yes
4883	pol (int), CTS	AAAAATTCAAAATTTTCGGGT	77.3			
4884	pol (int), CTS	AAAATTCAAAATTTTCGGGTT	76.7			
4885	pol (int), CTS	AAATTCAAAATTTTCGGGTTT	78.5			
4886	pol (int), CTS	AATTCAAAATTTTCGGGTTTA	78.7			
4887	pol (int), CTS	ATTCAAAATTTTCGGGTTTAT	79.3		yes	
4888	pol (int), CTS	TTCAAAATTTTCGGGTTTATT	80.6	yes		yes
4889	pol (int), CTS	TCAAAATTTTCGGGTTTATTA	76.9		yes	
4890	pol (int), CTS	CAAAATTTTCGGGTTTATTAC	77.5			
4891	pol (int), CTS	AAAATTTTCGGGTTTATTACA	77.7			
4892	pol (int), CTS	AAATTTTCGGGTTTATTACAG	78.5			
4953	pol (int)	CTCTGGAAAGGTGAAGGGGCA	82.2			yes
4954	pol (int)	TCTGGAAAGGTGAAGGGGCAG	82.0			
4955	pol (int)	CTGGAAAGGTGAAGGGGCAGT	82.2			yes
4956	pol (int)	TGGAAAGGTGAAGGGGCAGTA	84.0			
4957	pol (int)	GGAAAGGTGAAGGGGCAGTAG	83.6			
4958	pol (int)	GAAAGGTGAAGGGGCAGTAGT	83.4			
4959	pol (int)	AAAGGTGAAGGGGCAGTAGTA	72.1		yes	yes
4961	pol (int)	AGGTGAAGGGGCAGTAGTAAT	70.1	yes		yes
5040	pol (int), vif	TATGGAAAACAGATGGCAGGT	90.7			
5041	pol (int), vif	ATGGAAAACAGATGGCAGGTG	90.1			
5966	pol (int), vif	TCCTATGGCAGGAAGAAGCGG	81.5			
5967	pol (int), vif	CCTATGGCAGGAAGAAGCGGA	79.9			

HXB2 coordinate	Target gene	Target site (21 bp)	Conservation (%)	siRNA efficacy prediction Ui-Tei	Reynolds	Amarzguioui
5970	pol (int), vif	ATGGCAGGAAGAAGCGGAGAC	70.2			
7654	env (gp120)	ACAATTGGAGAAGTGAATTAT	70.7			
7655	env (gp120)	CAATTGGAGAAGTGAATTATA	70.5		yes	
7796	env (gp41)	AGCAGCAGGAAGCACTATGGG	71.1			
7797	env (gp41)	GCAGCAGGAAGCACTATGGGC	71.7			
7798	env (gp41)	CAGCAGGAAGCACTATGGGCG	75.4			
7799	env (gp41)	AGCAGGAAGCACTATGGGCGC	75.8			
7830	env (gp41)	ACGCTGACGGTACAGGCCAGA	72.1			yes
7831	env (gp41)	CGCTGACGGTACAGGCCAGAC	72.9			
9063	nef, 3'PPT	CTTTTTAAAAGAAAAGGGGGG	86.1			
9064	nef, 3'PPT	TTTTTAAAAGAAAAGGGGGGA	89.5			
9065	nef, 3'PPT	TTTTAAAAGAAAAGGGGGGAC	90.2			
9066	nef, 3'PPT, U3	TTTAAAAGAAAAGGGGGGACT	90.1			
9067	nef, 3'PPT, U3	TTAAAAGAAAAGGGGGGACTG	90.3			
9068	nef, 3'PPT, U3	TAAAAGAAAAGGGGGGACTGG	90.5			
9069	nef, 3'PPT, U3	AAAAGAAAAGGGGGGACTGGA	90.7			
