# Supplementary figures and images for: Optimal design and validation of antiviral siRNA for targeting HIV-1
Source: Retrovirology. 2007 Nov 8;4:80. doi: 10.1186/1742-4690-4-80 (PMC2204037; doi:10.1186/1742-4690-4-80)

siRNA sequences

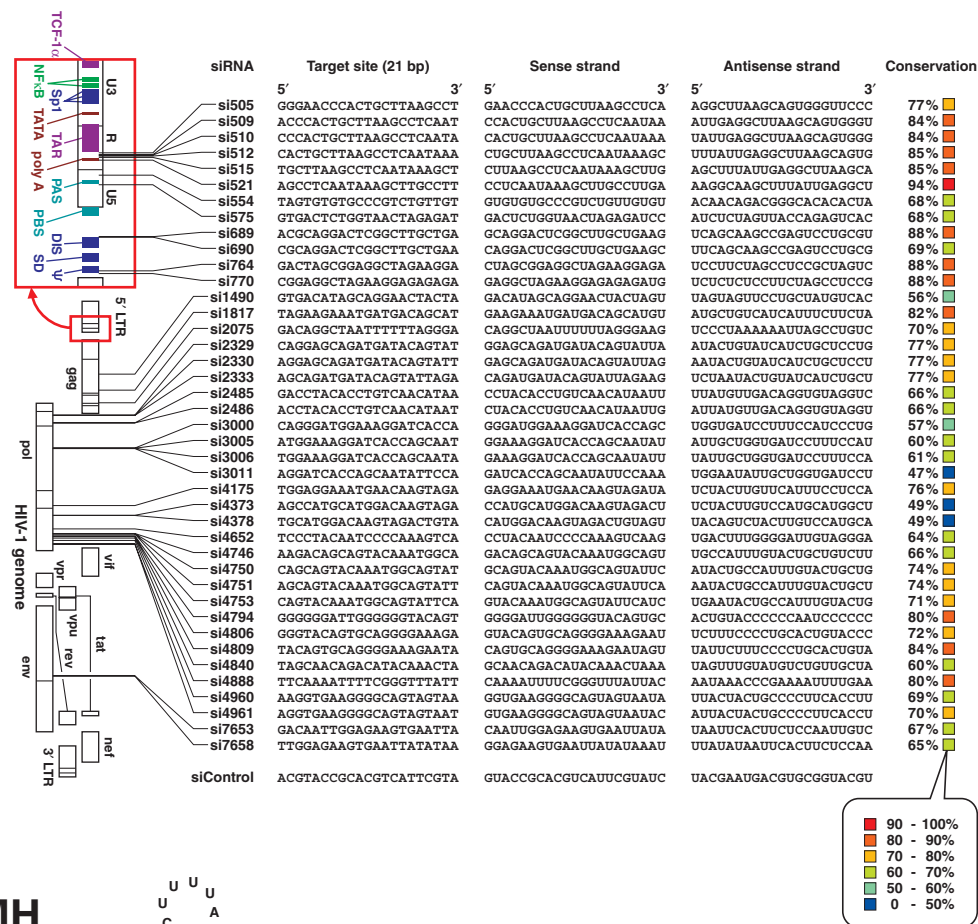

BMH

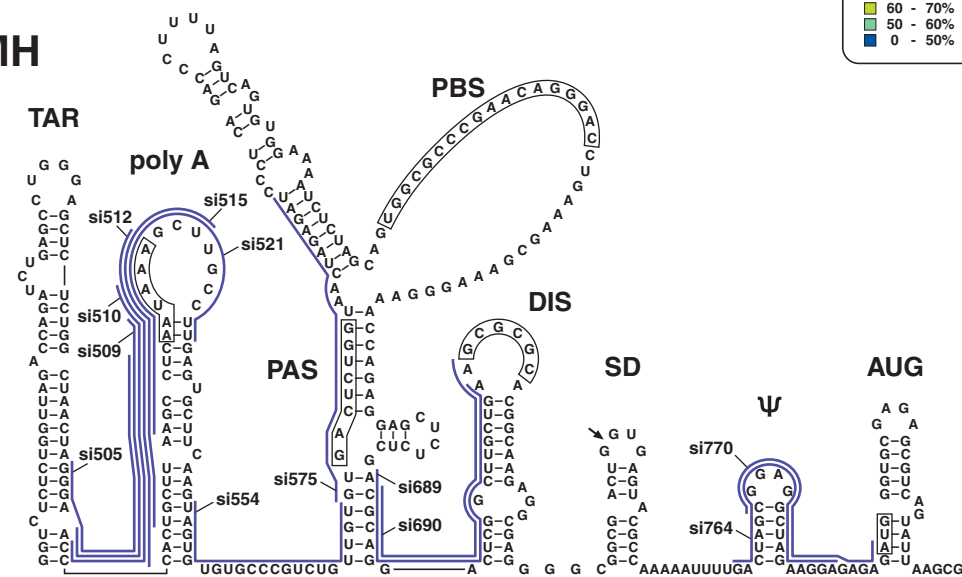

LDI

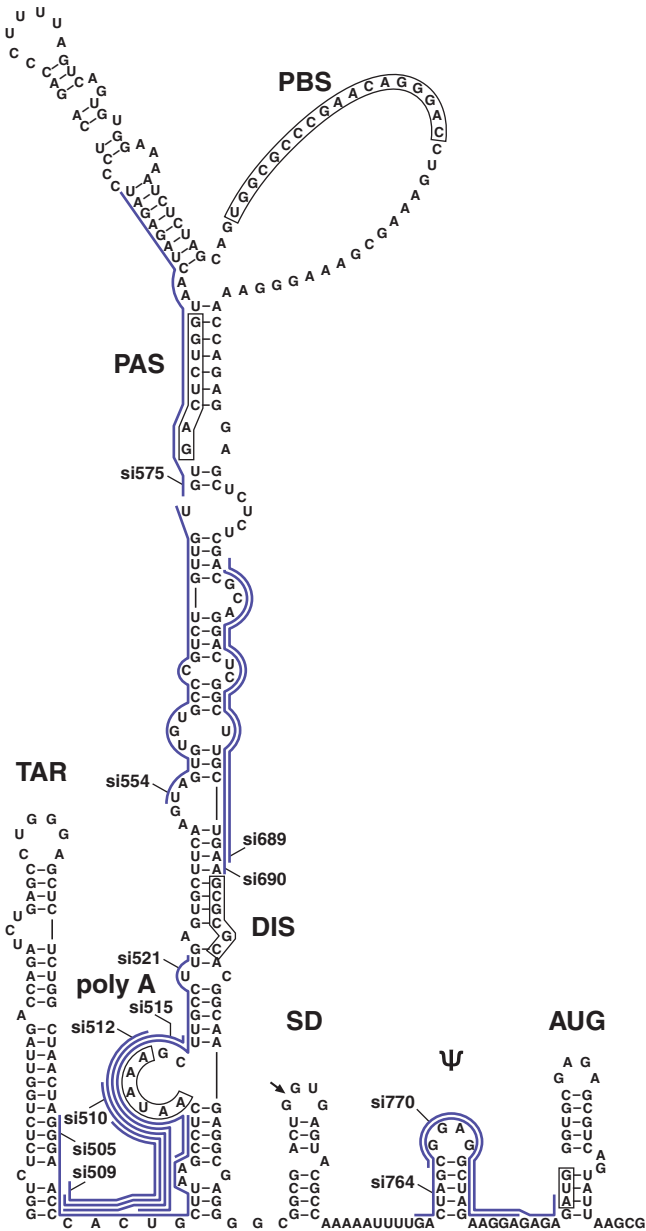

Supplement: Additional file 4 — The siRNA sequences and their target sites. The sequences of 41 siRNAs and their target sites are shown. The siRNA numbers indicate the nucleotide position in HXB2 (GenBank K03455). The conservation level of each siRNA in HIV-1 group M sequence is depicted in color chart at the rightmost column. BMH (branched multiple hairpin) and LDI (long distance interaction) conformations of the HIV-1 leader RNA and siRNAs targeting them are shown. [file 1742-4690-4-80-S4.pdf]

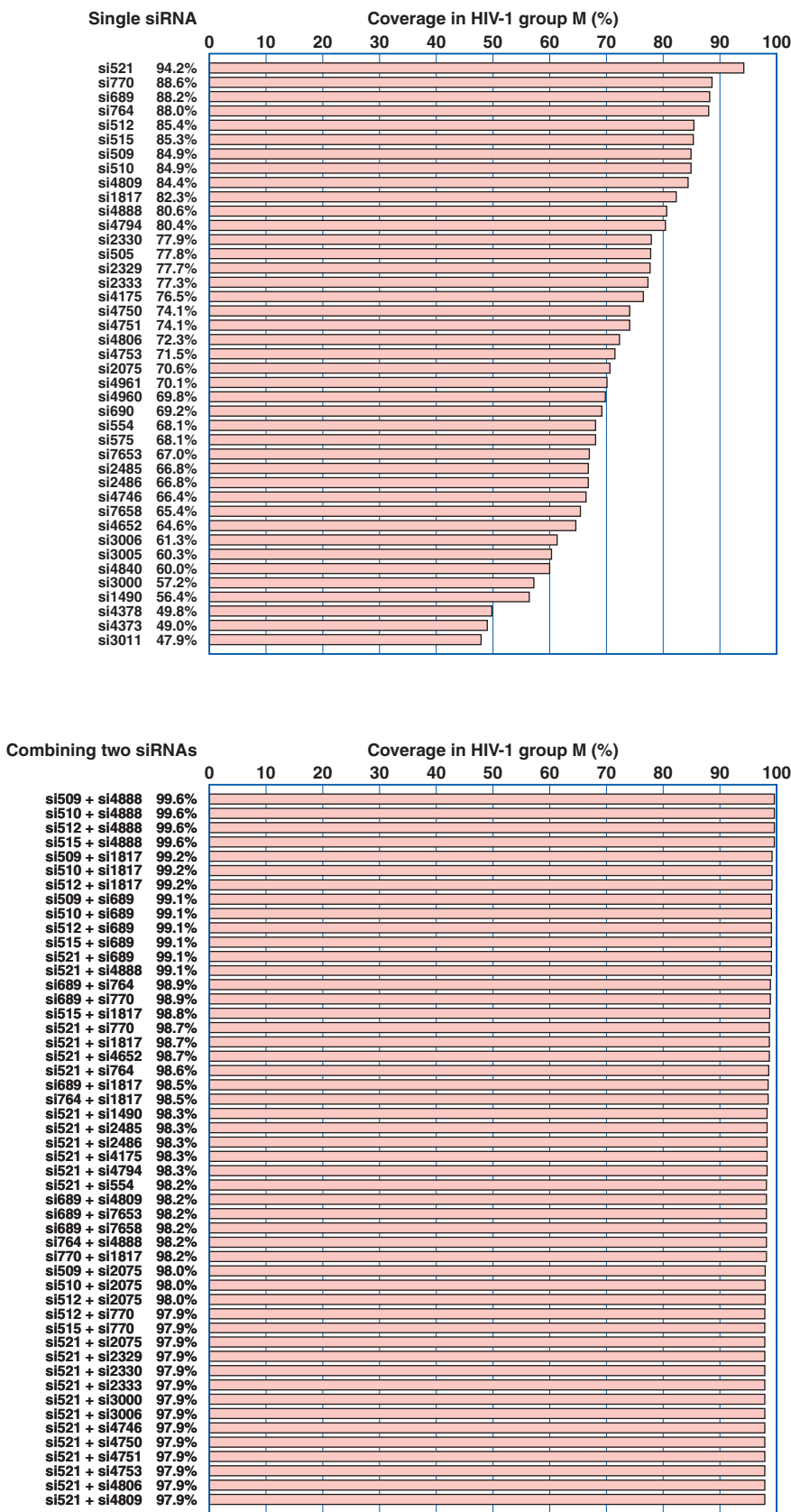

Supplement: Additional file 7 — Coverage of HIV-1 group M by single siRNA or two siRNAs. (A) Coverage of HIV-1 group M by 41 siRNAs used in this study. (B) Coverage of HIV-1 group M by combining two siRNAs from above. Coverage was calculated by considering only the HIV-1 sequences which contain the corresponding regions. [file 1742-4690-4-80-S7.pdf]
