# Supplementary material for: Optimal design and validation of antiviral siRNA for targeting HIV-1
Source: Retrovirology. 2007 Nov 8;4:80. doi: 10.1186/1742-4690-4-80 (PMC2204037; doi:10.1186/1742-4690-4-80)
Supplement: Additional file 5 — Supplementary materials and methods. [file 1742-4690-4-80-S5.pdf]

siRNAs. Chemically synthesized siRNA duplexes were purchased from RNAi Co., Ltd. (Tokyo, Japan). The siRNA sequences are detailed in **Additional file 4**. HIV-1 specific siRNAs were numbered according to the nucleotide position in HXB2 (GenBank K03455).

Cell culture and transfection. HeLa cells were cultured in Dulbecco's modified Eagle medium (DMEM; Gibco) supplemented with 10% heat-inactivated fetal bovine serum (Mitsubishi Chemical), 10 U/ml penicillin, and 50 μg/ml streptomycin. Transfections were performed in 24-well plates using Lipofectamine 2000 (Invitrogen) according to the manufacturer's protocol.

Target mRNA cleavage assay. We developed CMV-driven target expression vector pTREC, which is a derivative of pCI-neo, for expressing various target sequences within mammalian cells (Ui-Tei K *et al.*, *Methods Mol Biol* 2006, **361:**201-216). To test whether each siRNA is functional, 23-39 bp target sequence was made by synthetic DNA oligonucleotide duplex and inserted into EcoRI and XhoI sites of the pTREC vector. The resulting pTREC constructs were cotransfected into HeLa cells (0.5 μg/well) with their corresponding siRNAs (5 nM). Cells were harvested 24 h after the transfection and total RNA was isolated using RNeasy 96 (Qiagen). cDNA was synthesized using SuperScript II reverse transcriptase (Invitrogen) and oligo dT primer. The quantity of the target mRNA was normalized by simultaneously expressed *neo* gene. Quantitative real-time RT-PCR was performed by ABI PRISM 7000 (Applied Biosystems) using SYBR green PCR master mix (Applied Biosystems). PCR primer sequences were as follows: target mRNA, 5′-AGG CAC TGG GCA GGT GTC and 5′-TGC TCG AAG CAT TAA CCC TCA CTA; and *neo* (internal control), 5′-ATC AGG ATG ATC TGG ACG AAG and 5′-CTC TTC AGC AAT ATC ACG GGT.

Viral reverse transcriptase activity assay. Each siRNA was cotransfected into HeLa cells at 5 nM with one of the four infectious molecular clones, pNL4-3 (subtype B: GenBank M19921), 95MM-yIDU106 (subtype B′, Thailand variant of subtype B: Takebe, Y. *et al.*, unpublished), 93IN101 (subtype C: GenBank AB023804) or 93JP-NH1 (CRF01_AE: GenBank AB052995). Molecular clone was transfected at 0.2 μg/well (pNL4-3) or at 1 μg/well (95MM-yIDU106, 93IN101 and 93JP-NH1). Culture supernatants were collected 48 h after the transfection and viral reverse transcriptase activity was measured as previously described (Willey RL *et al.*, *J Virol* 1988, **62:**139-147).
