# Supplementary material for: Optimal design and validation of antiviral siRNA for targeting HIV-1
Source: Retrovirology. 2007 Nov 8;4:80. doi: 10.1186/1742-4690-4-80 (PMC2204037; doi:10.1186/1742-4690-4-80)
Supplement: Additional file 6 — Target sites of the 41 siRNAs used in this study. Sequence alignment of the target site from the four HIV-1 infectious molecular clones: pNL4-2 (subtype B); 95MM-yIDU106 (subtype B'); 93IN101 (subtype C); or 93JP-NH1 (CRF01_AE). [file 1742-4690-4-80-S6.pdf]

Additional file 6 (Naito, Y. *et al.*)


```
si505         GGGAACCCACTGCTTAAGCCT
pNL4-3        .....................
95MM-yIDU106  .....................
93IN101       .....................
93JP-NH1      .................AG.C

si509         ACCCACTGCTTAA-GCCTCAAT
pNL4-3        .............-........
95MM-yIDU106  .............-........
93IN101       .............-........
93JP-NH1      .............A........

si510         CCCACTGCTTAA-GCCTCAATA
pNL4-3        ............-.........
95MM-yIDU106  ............-.........
93IN101       ............-.........
93JP-NH1      ............A.........

si512         CACTGCTTAA-GCCTCAATAAA
pNL4-3        ..........-...........
95MM-yIDU106  ..........-...........
93IN101       ..........-...........
93JP-NH1      ..........A...........

si515         TGCTTAA-GCCTCAATAAAGCT
pNL4-3        .......-..............
95MM-yIDU106  .......-..............
93IN101       .......-..............
93JP-NH1      .......A..............

si521         AGCCTCAATAAAGCTTGCCTT
pNL4-3        .....................
95MM-yIDU106  .....................
93IN101       .....................
93JP-NH1      .....................

si554         TAGTGTGTGCCCGTCTGTTGT
pNL4-3        .....................
95MM-yIDU106  ....A................
93IN101       C...............A....
93JP-NH1      .G................GT.

si575         GTGACTCTGGTAACTAGAGAT
pNL4-3        .....................
95MM-yIDU106  ............G........
93IN101       .....................
93JP-NH1      AG...................

si689         ACGCAGGACTCGGCTTGCTGA
pNL4-3        .....................
95MM-yIDU106  .....................
93IN101       .....................
93JP-NH1      .....................

si690         CGCAGGACTCGGCTTGCTGAA
pNL4-3        .....................
95MM-yIDU106  .....................
93IN101       .....................
93JP-NH1      ....................G

si764         GACTAGCGGAGGCTAGAAGGA
pNL4-3        .....................
95MM-yIDU106  .....................
93IN101       .....................
93JP-NH1      .....................

si770         CGGAGGCTAGAAGGAGAGAGA
pNL4-3        .....................
95MM-yIDU106  .....................
93IN101       .....................
93JP-NH1      .....................

si1490        GTGACATAGCAGGAACTACTA
pNL4-3        .....................
95MM-yIDU106  .....................
93IN101       .....................
93JP-NH1      .....................

si1817        TAGAAGAAATGATGACAGCAT
pNL4-3        .....................
95MM-yIDU106  .....................
93IN101       .......G.............
93JP-NH1      .....................

si2075        GACAGGCTAATTTTTTAGGGA
pNL4-3        .....................
95MM-yIDU106  .....................
93IN101       .G...................
93JP-NH1      .....................

si2329        CAGGAGCAGATGATACAGTAT
pNL4-3        .....................
95MM-yIDU106  .....................
93IN101       .....................
93JP-NH1      .....................

si2330        AGGAGCAGATGATACAGTATT
pNL4-3        .....................
95MM-yIDU106  .....................
93IN101       .....................
93JP-NH1      .....................

si2333        AGCAGATGATACAGTATTAGA
pNL4-3        .....................
95MM-yIDU106  .....................
93IN101       .....................
93JP-NH1      .....................

si2485        GACCTACACCTGTCAACATAA
pNL4-3        .....................
95MM-yIDU106  .....................
93IN101       ....C......A.........
93JP-NH1      .....................

si2486        ACCTACACCTGTCAACATAAT
pNL4-3        .....................
95MM-yIDU106  .....................
93IN101       ...C......A..........
93JP-NH1      .....................

si3000        CAGGGATGGAAAGGATCACCA
pNL4-3        .....................
95MM-yIDU106  .....................
93IN101       .....................
93JP-NH1      .....................

si3005        ATGGAAAGGATCACCAGCAAT
pNL4-3        .....................
95MM-yIDU106  .....................
93IN101       .....................
93JP-NH1      .....................

si3006        TGGAAAGGATCACCAGCAATA
pNL4-3        .....................
95MM-yIDU106  .....................
93IN101       .....................
93JP-NH1      .....................

si3011        AGGATCACCAGCAATATTCCA
pNL4-3        .....................
95MM-yIDU106  .....................
93IN101       .....................
93JP-NH1      .....................

si4175        TGGAGGAAATGAACAAGTAGA
pNL4-3        .....................
95MM-yIDU106  ......G.........A....
93IN101       .....................
93JP-NH1      .....................

si4373        AGCCATGCATGGACAAGTAGA
pNL4-3        .....................
95MM-yIDU106  .....................
93IN101       .....................
93JP-NH1      .....................

si4378        TGCATGGACAAGTAGACTGTA
pNL4-3        .....................
95MM-yIDU106  .....................
93IN101       .....................
93JP-NH1      .....................

si4652        TCCCTACAATCCCCAAAGTCA
pNL4-3        .....................
95MM-yIDU106  .....................
93IN101       .....................
93JP-NH1      C....................

si4746        AAGACAGCAGTACAAATGGCA
pNL4-3        .....................
95MM-yIDU106  .....................
93IN101       .....................
93JP-NH1      .....................

si4750        CAGCAGTACAAATGGCAGTAT
pNL4-3        .....................
95MM-yIDU106  .....................
93IN101       .....................
93JP-NH1      .....................

si4751        AGCAGTACAAATGGCAGTATT
pNL4-3        .....................
95MM-yIDU106  .....................
93IN101       .....................
93JP-NH1      .....................

si4753        CAGTACAAATGGCAGTATTCA
pNL4-3        .....................
95MM-yIDU106  .....................
93IN101       .....................
93JP-NH1      ...................T.

si4794        GGGGGGATTGGGGGGTACAGT
pNL4-3        .....................
95MM-yIDU106  .............AC...T..
93IN101       .....................
93JP-NH1      .....................

si4806        GGGTACAGTGCAGGGGAAAGA
pNL4-3        .....................
95MM-yIDU106  .AC...T..............
93IN101       .....................
93JP-NH1      .....................

si4809        TACAGTGCAGGGGAAAGAATA
pNL4-3        .....................
95MM-yIDU106  ...T.................
93IN101       .....................
93JP-NH1      .....................

si4840        TAGCAACAGACATACAAACTA
pNL4-3        .....................
95MM-yIDU106  .....................
93IN101       .....................
93JP-NH1      .....................

si4888        TTCAAAATTTTCGGGTTTATT
pNL4-3        .....................
95MM-yIDU106  .....................
93IN101       .....................
93JP-NH1      .....................

si4960        AAGGTGAAGGGGCAGTAGTAA
pNL4-3        .....................
95MM-yIDU106  .....................
93IN101       .....................
93JP-NH1      .....................

si4961        AGGTGAAGGGGCAGTAGTAAT
pNL4-3        .....................
95MM-yIDU106  .....................
93IN101       .....................
93JP-NH1      .....................

si7653        GACAATTGGAGAAGTGAATTA
pNL4-3        .....................
95MM-yIDU106  .....................
93IN101       .....................
93JP-NH1      .....................

si7658        TTGGAGAAGTGAATTATATAA
pNL4-3        .....................
95MM-yIDU106  .....................
93IN101       .....................
93JP-NH1      .....................
```

	GenBank	Subtype/CRF
**pNL4-3**	M19921	subtype B
**95MM-yIDU106**	Takebe, Y. *et al.* (unpublished)	subtype B′
**93IN101**	AB023804	subtype C
**93JP-NH1**	AB052995	CRF01_AE
